# A Trio of Viral Proteins Tunes Aphid-Plant Interactions in *Arabidopsis thaliana*

**DOI:** 10.1371/journal.pone.0083066

**Published:** 2013-12-11

**Authors:** Jack H. Westwood, Simon C. Groen, Zhiyou Du, Alex M. Murphy, Damar Tri Anggoro, Trisna Tungadi, Vijitra Luang-In, Mathew G. Lewsey, John T. Rossiter, Glen Powell, Alison G. Smith, John P. Carr

**Affiliations:** 1 Department of Plant Sciences, University of Cambridge, Cambridge, United Kingdom; 2 Imperial College, London, United Kingdom; University of California, Riverside, United States of America

## Abstract

**Background:**

Virus-induced deterrence to aphid feeding is believed to promote plant virus transmission by encouraging migration of virus-bearing insects away from infected plants. We investigated the effects of infection by an aphid-transmitted virus, cucumber mosaic virus (CMV), on the interaction of *Arabidopsis thaliana*, one of the natural hosts for CMV, with *Myzus persicae* (common names: ‘peach-potato aphid’, ‘green peach aphid’).

**Methodology/Principal Findings:**

Infection of Arabidopsis (ecotype Col-0) with CMV strain Fny (Fny-CMV) induced biosynthesis of the aphid feeding-deterrent 4-methoxy-indol-3-yl-methylglucosinolate (4MI3M). 4MI3M inhibited phloem ingestion by aphids and consequently discouraged aphid settling. The CMV 2b protein is a suppressor of antiviral RNA silencing, which has previously been implicated in altering plant-aphid interactions. Its presence in infected hosts enhances the accumulation of CMV and the other four viral proteins. Another viral gene product, the 2a protein (an RNA-dependent RNA polymerase), triggers defensive signaling, leading to increased 4MI3M accumulation. The 2b protein can inhibit ARGONAUTE1 (AGO1), a host factor that both positively-regulates 4MI3M biosynthesis and negatively-regulates accumulation of substance(s) toxic to aphids. However, the 1a replicase protein moderated 2b-mediated inhibition of AGO1, ensuring that aphids were deterred from feeding but not poisoned. The LS strain of CMV did not induce feeding deterrence in Arabidopsis ecotype Col-0.

**Conclusions/Significance:**

Inhibition of AGO1 by the 2b protein could act as a booby trap since this will trigger antibiosis against aphids. However, for Fny-CMV the interplay of three viral proteins (1a, 2a and 2b) appears to balance the need of the virus to inhibit antiviral silencing, while inducing a mild resistance (antixenosis) that is thought to promote transmission. The strain-specific effects of CMV on Arabidopsis-aphid interactions, and differences between the effects of Fny-CMV on this plant and those seen previously in tobacco (inhibition of resistance to aphids) may have important epidemiological consequences.

## Introduction

Viruses induce extensive biochemical changes in plants [1]. These changes can affect interactions of plants with the vectors of viruses and may influence transmission of viruses from infected plants to new hosts [2–4]. This may be particularly true for viruses that are transmitted by aphids, which are the most prevalent vectors of plant-infecting viruses [4]. In the ‘non-persistent’ mode of aphid-mediated virus transmission, which is the most commonly occurring form, virus particles bind to receptors present in the specialized mouthparts (stylet) of the insects [5]. When an aphid feeds on an infected plant, the attachment of virus particles to these receptors occurs within seconds [6]. Thus, virus acquisition does not require prolonged feeding from vascular tissues; virus particles are acquired most efficiently as the aphid tests the plant for palatability by brief probe feeds from the epidermal cells and these cells are also the primary inoculation sites during aphid-mediated infection [6]. However, virus particles are very weakly bound to the stylet and are easily dislodged during salivation, which will occur inevitably if feeding is prolonged [6]. For these reasons, prolonged settling and feeding from the phloem by aphids is thought to diminish their effectiveness as vectors for non-persistently transmitted viruses [3,4]. The induction of aphid feeding deterrence in plant hosts following virus infection has been proposed as a mechanism by which viruses could promote their own transmission [4]. Indeed, an exhaustive meta-analysis of the literature in this area suggested a significant trend for the evolution of viruses towards promoting these transmission-enhancing changes in plants [4].

However, the effects of a virus on host plant biochemistry can affect aphid species differentially. For example, on potato plants infected with the potyvirus potato virus Y (PVY), feeding by the aphid *Macrosiphum euphorbiae* was inhibited (consistent with encouragement of transmission), whereas feeding by *Myzus persicae* was enhanced, which is less likely to encourage PVY transmission by members of this aphid species [7]. There are also host-specific aspects to virus-plant-vector interactions. For example, Mauck and colleagues [8] observed that squash (*Cucurbita pepo*) infected with the Fny strain of cucumber mosaic virus (Fny-CMV) emitted increased levels of volatile compounds that attracted aphids but that the same plants became distasteful to the insects. Since aphids transmit CMV *via* the non-persistent mode, these authors proposed that the combination of increased attractiveness and feeding deterrence would serve to increase transmission of the virus [8]. By contrast, also using Fny-CMV, we found that in tobacco the virus did not induce resistance to feeding by *M. persicae* and that it may suppress the induction of resistance to aphids [9]. These contrasting results obtained with CMV lend further credence to the idea that viruses have host-specific effects on aphid-plant interactions; in some hosts inducing resistance to settling, which will enhance transmission, whilst in other hosts fostering aphid survival.

Unfortunately, hosts such as tobacco, potato or squash do not lend themselves to detailed dissection of the complex molecular processes linking virus infection to changes in the aphid-plant relationship. Therefore, focusing specifically on viral effects on aphid growth and feeding behavior, we investigated the effects of two aphid-transmissible CMV strains, Fny-CMV and LS-CMV [10], on aphid-plant interactions in *Arabidopsis thaliana* (hereafter referred to as Arabidopsis). This plant is not only a well-studied genetic model but is also a very common natural host for CMV in the wild [11].

## Results

### CMV induced resistance to the aphid *Myzus persicae*

**Figure 1:**
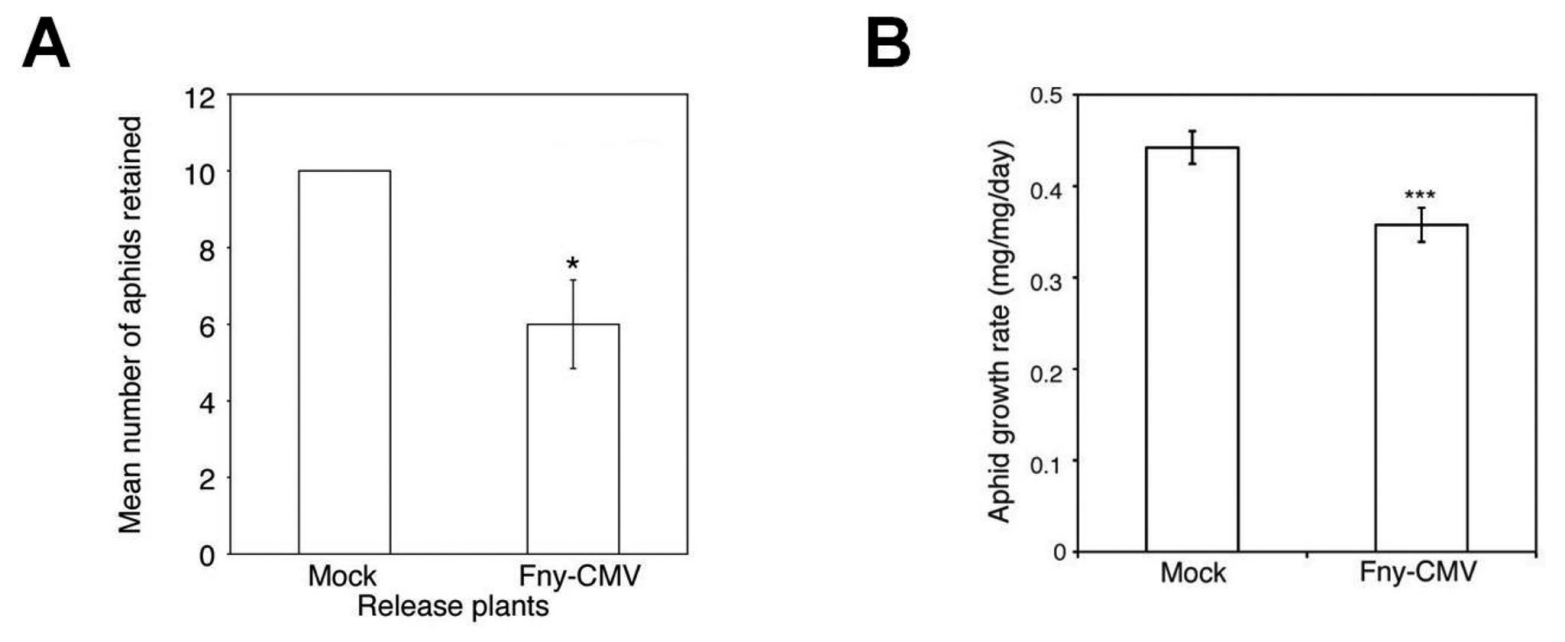
Aphid behavior and performance on virus-infected wild-type Arabidopsis plants. (A) Ten aphids (*Myzus persicae*) were released onto rosettes of mock-inoculated or Fny-CMV-infected release plants and then allowed to remain or emigrate to a plant of the opposite treatment group located 10 cm away in the same pot. Aphids migrated away more often from Fny-CMV-infected than from mock-inoculated plants. Fewer aphids remained on Fny-CMV-infected than on mock-inoculated release plants after 24 hours. Based on the methods of Mauck et al. [8], three independent tests were performed for each type of release plant. See Figure S1 for the accompanying aphid choice data. (B) Mean relative growth rate of individual aphids feeding on Arabidopsis plants infected with Fny-CMV, n≥24. Error bars represent standard error of the mean. Asterisks indicate significant differences (Student’s *t*-test): *, *P*<0.05; **, *P*<0.01; ***, *P*<0.001.

**Figure 2:**
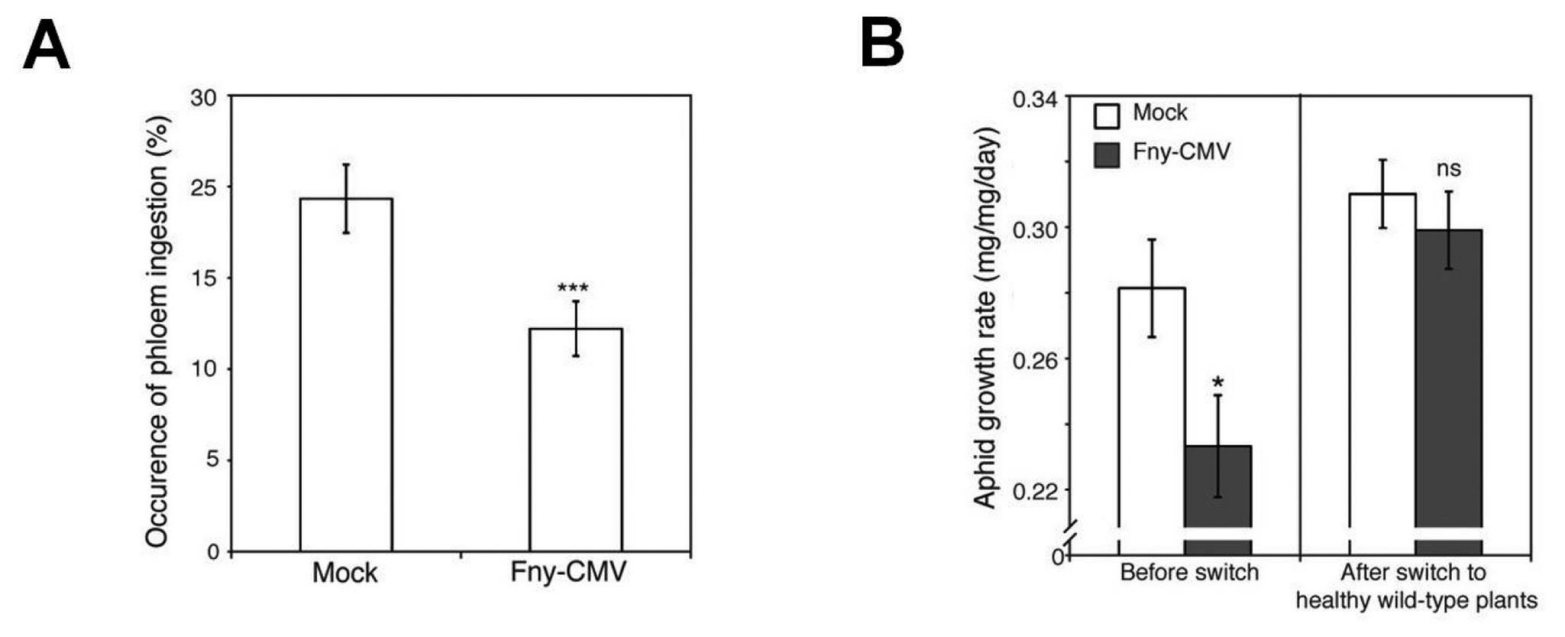
Aphid behavior and performance on virus-infected wild-type Arabidopsis plants. (A) Electrical penetration graph analysis of aphid feeding behavior over 12-hour periods revealed that aphids were less likely to feed from the phloem of Fny-CMV-infected plants, n=15. (B) When aphids previously fed on Fny-CMV-infected plants were moved to uninfected wild-type plants, their growth rate recovered to levels similar to those fed on uninfected plants, n≥24. Error bars represent standard error of the mean. Asterisks indicate significant differences (Student’s *t*-test): *, *P*<0.05; **, *P*<0.01; ***, *P*<0.001. Non-significant: ns.

Aphids (*M. persicae*) migrated away from Arabidopsis (ecotype Col-0) plants infected with CMV (strain Fny) (Figure 1A, Figure S1). While investigating the nature of this resistance, we found that aphids confined on infected plants grew less well than on mock-inoculated plants (Figure 1B), that they took longer to reproduce, and that they gave rise to smaller colonies (Figures S2A, S2B). Electrical penetration graph (EPG) [12] measurements showed that aphids on Fny-CMV-infected plants ingested less phloem sap, which would normally be their major nutrition source (Figure 2A, Figure S3). This indicated that feeding deterrence, not host toxicity, inhibited the growth of aphids confined on Fny-CMV-infected plants. We confirmed this by showing that when aphids that were initially confined on Fny-CMV-infected hosts were transferred to healthy plants, they recovered and began to grow at normal rates (Figure 2B).

### Fny-CMV triggered defense-related plant gene expression and changes in secondary metabolism

Microarray analysis indicated that altered host gene expression might underpin Fny-CMV-induced resistance to *M. persicae*. Fny-CMV induced significant expression changes for 920 genes (Spreadsheet S1). CMV infection induces salicylic acid (SA) accumulation and changes in gene expression in the Col-0 ecotype of Arabidopsis. This is despite the fact that these plants are susceptible to CMV and that the virus induces no hypersensitive response in this host [13,14]. Our gene ontology analysis highlighted many defense- and SA-related transcriptional changes (Spreadsheets S1, S2). Among the SA-responsive transcripts most affected by Fny-CMV infection were *ISOCHORISMATE SYNTHASE1* (*ICS1*) [15], SA-responsive *PATHOGENESIS-RELATED PROTEIN1* and -5, and *SENESCENCE-ASSOCIATED GENE13* and -21 (Figure S4) (Spreadsheet S1). Although Fny-CMV increased SA-responsive gene expression, SA probably does not promote resistance to aphids; indeed, for certain phloem-feeders (whiteflies) this phytohormone facilitates infestation [16].

**Figure 3:**
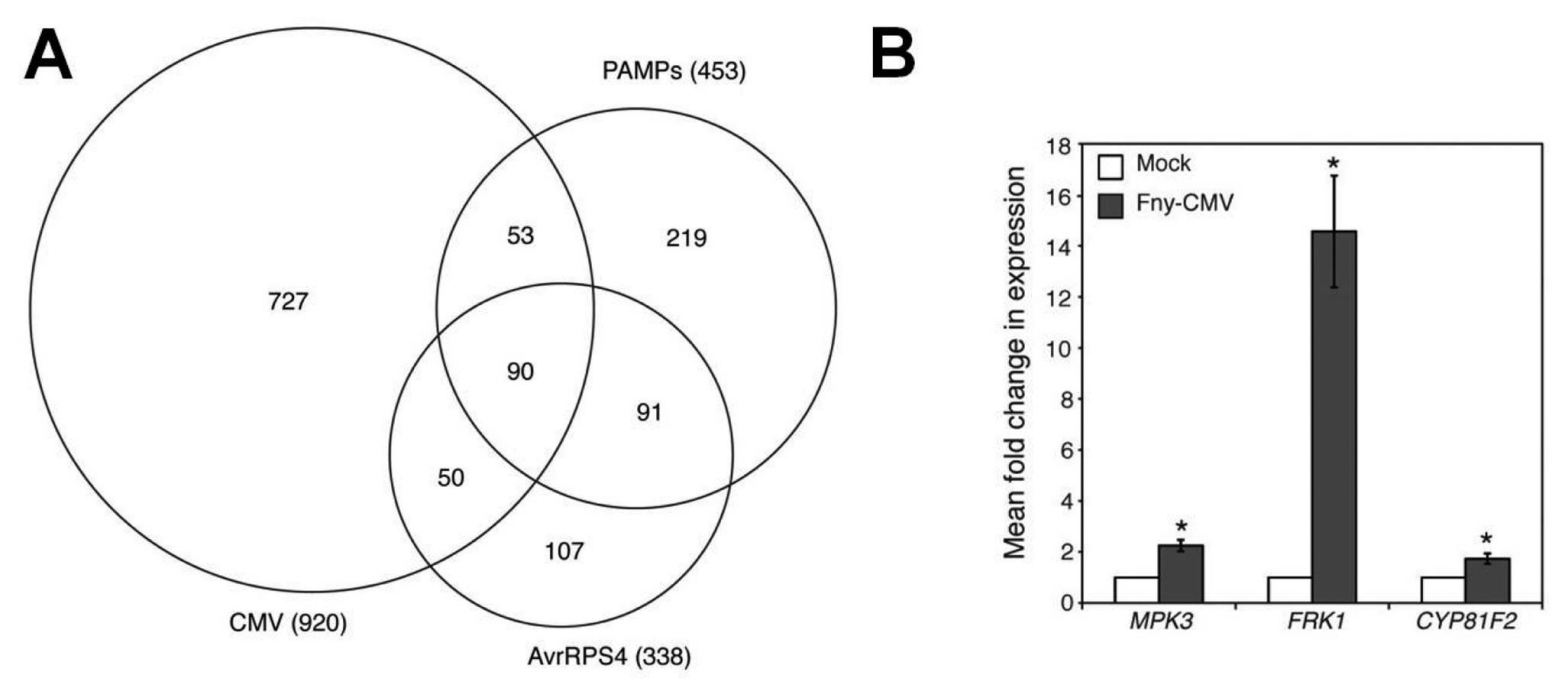
Fny-CMV infection of Arabidopsis induced genes that are also activated during PAMP- and effector-triggered immunity. (A) Area-proportional Venn diagram showing overlap between genes activated during PAMP- and effector-triggered immunity. Genes up-regulated by Fny-CMV infection were compared with previously published microarray data showing genes commonly induced by three PAMPs (flg22, elf26, and chitin) [84], and genes induced by the *Pseudomonas syringae* effector AvrRPS4 in RPS4 Col-0 Arabidopsis [85]. Venn diagram was drawn based on an image generated using a free online program (http://bioinforx.com/free/bxarrays/venndiagram.php). (B) Accumulation of the defense-related transcripts MPK3, FRK1 and *CYP81F2* was induced following infection with Fny-CMV as measured by RT-Q-PCR. Error bars represent standard error of the mean. Asterisks indicate significant differences (Student’s *t*-test): *, *P*<0.05.

**Figure 4:**
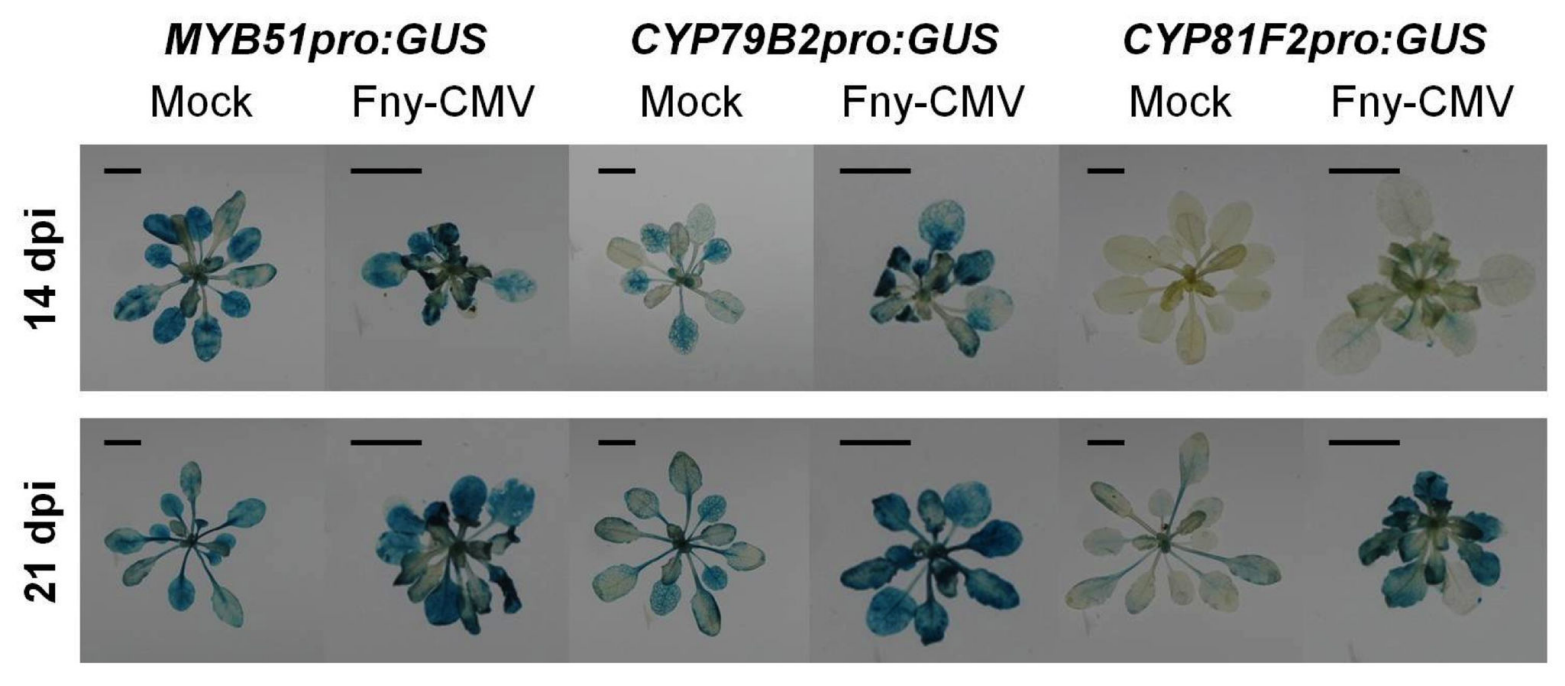
Induction of *GUS* reporter genes fused to the promoters of MYB51*, CYP79B2*, and *CYP81F2* in response to Fny-CMV infection at 14 and 21 days post-inoculation (dpi). Three plants per treatment group, per time-point were analyzed and photographs were taken of representative plants. Scale bars represent 1 cm.

Fny-CMV also induced genes known to be responsive to pathogen-associated molecular pattern (PAMP) molecules such as flg22, elf26 and chitin (Spreadsheet S2). PAMP-responsive transcripts affected by Fny-CMV included those for genes conditioning MPK3-dependent MAP kinase signaling, which orchestrates PAMP-triggered immunity (PTI) [17,18]. *MAPKKK10*, *MKK4* and *MPK3* were all significantly induced following infection (Spreadsheet S1). Meta-analysis of available microarray datasets revealed an overlap in up-regulation for 90 genes caused not only by Fny-CMV infection, but also by PAMPs and specific recognition of the bacterial effector AvrRPS4 (Figure 3A) (Spreadsheet S3). Reverse-transcription coupled to quantitative PCR (RT-Q-PCR) was used to confirm this and to detect induction of transcripts encoding MPK3 and factors downstream of MPK3 (FRK1 and CYP81F2) [18] (Figure 3B). Fny-CMV induced promoter-ß-glucuronidase (*GUS*) fusions for the promoters of *MYB51*, *CYP79B2*, and *CYP81F2* with the strongest signals being in the vascular tissue (Figure 4). This location is consistent with roles for these Fny-CMV-induced transcriptional changes in inhibition of aphid phloem feeding. Thus, Fny-CMV affects signaling elements shared by effector-triggered immunity (ETI) and PTI. Significantly, ETI and PTI coordinate defense against aphids. For example, avirulent bacteria trigger anti-aphid resistance [19] and *M. persicae* encodes effectors inhibiting flg22-induced PTI [20].

**Figure 5:**
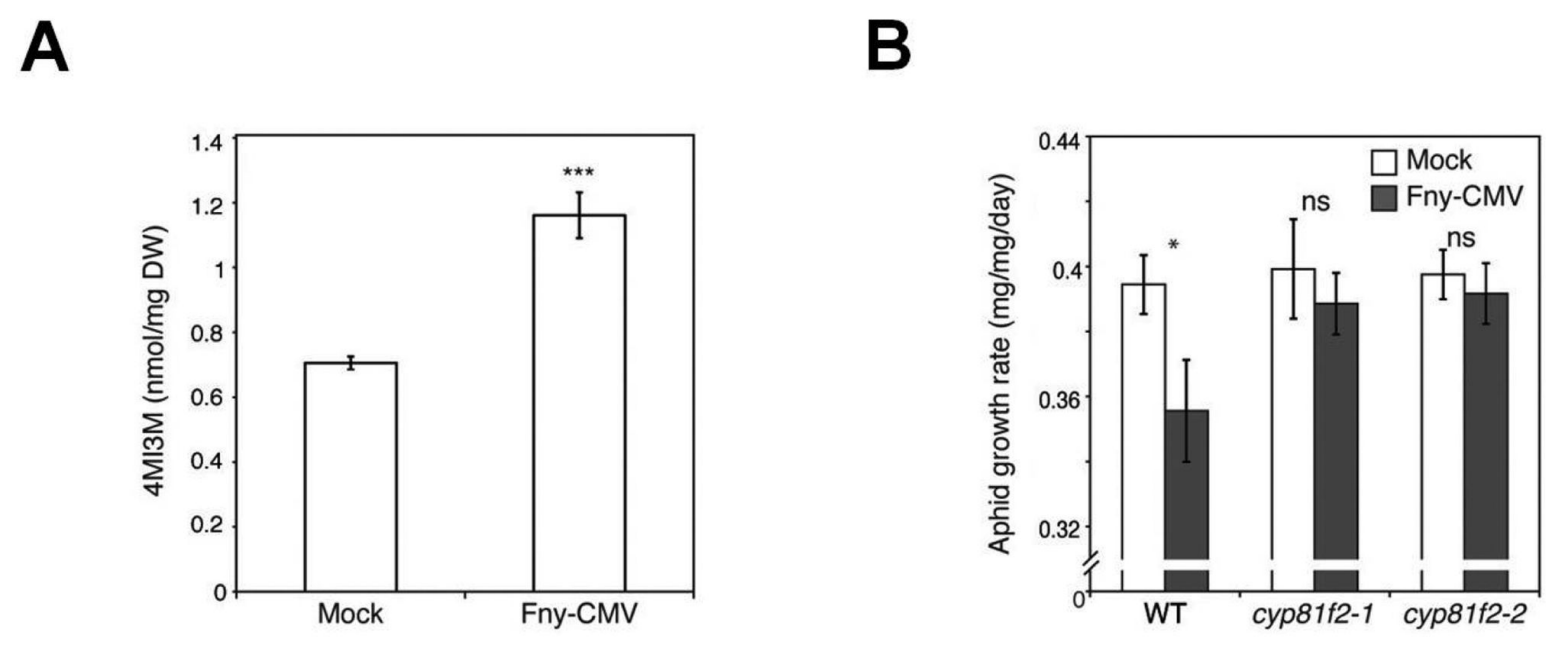
Fny-CMV infection of Arabidopsis induces resistance to aphids by inducing accumulation of 4-methoxy-indol3yl-methylglucosinolate (4MI3M). (A) High performance liquid chromatography analysis showed that Fny-CMV infection induced accumulation of the anti-feedant glucosinolate, 4MI3M. (B) Fny-CMV infection was unable to induce resistance in either of two independent *cyp81f2* mutant lines compromised in 4MI3M biosynthesis contrary to wild-type (WT) plants, n≥24. Error bars represent standard error of the mean. Asterisks indicate significant differences [Student’s *t*-test in (A), ANOVA with post-hoc Tukey’s tests in (B)]: *, *P*<0.05; **, *P*<0.01; ***, *P*<0.001. Non-significant: ns.

*CYP81F2* induction was notable because this gene encodes one of the critical enzymes in biosynthesis of the aphid feeding-deterrent 4-methoxy-indol-3-yl-methylglucosinolate (4MI3M) [21,22]. Consistent with this, analysis using high performance liquid chromatography (HPLC) showed that Fny-CMV infection triggered accumulation of 4MI3M (Figure 5A) and other glucosinolates (Figure S5). Using two independent 4MI3M-depleted *cyp81f2* mutant lines [23] we found that Fny-CMV-induced anti-aphid resistance relied on CYP81F2 function (Figure 5B). Clay and co-workers previously showed 4MI3M to be necessary for callose deposition [23], which could reduce phloem flow and inhibit aphid feeding [24]. However, we observed no reduction in virus-induced anti-aphid resistance on *pmr4-1* mutant plants (Figure S6), which are deficient in callose deposition [25]. Thus, 4MI3M or its breakdown products act as feeding deterrents [21] and provide the mechanism responsible for Fny-CMV-induced aphid resistance.

Recently, another secondary metabolite of Arabidopsis, camalexin, was suggested to mediate aphid resistance [26]. HPLC analysis showed that camalexin levels increased in Fny-CMV-infected plants (Figure S7A, S7B) but experiments with *pad3-1* mutant plants (which are compromised in camalexin biosynthesis [27]) showed that camalexin plays little or no role in the resistance to aphids induced by Fny-CMV (Figure S7C). 

### Identification of CMV-encoded factors affecting Arabidopsis-*M. persicae* interactions

**Figure 6:**
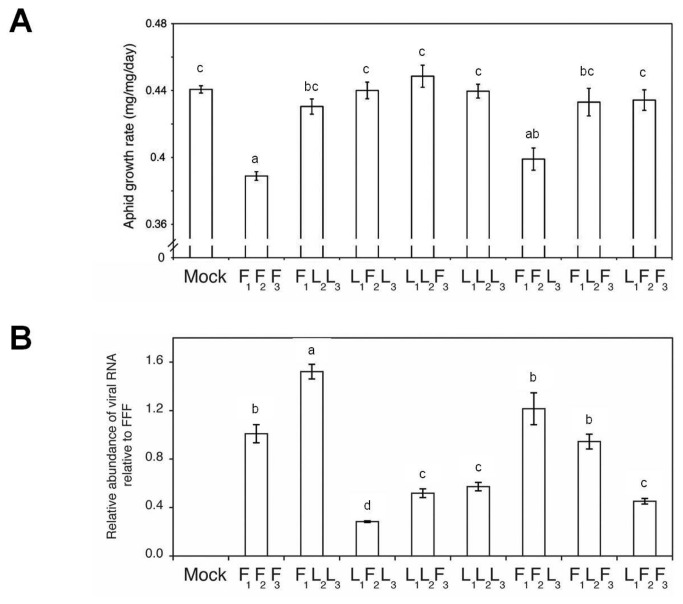
Virus-induced anti-aphid resistance mapped to Fny-CMV RNA2. (A) Growth rate of individual aphids on plants infected with reassortant versions of CMV, n≥24. (B) Accumulation of viral RNA measured by RT-Q-PCR relative to the accumulation of Fny-CMV (F_1_F_2_F_3_) at 14 days post-inoculation. Viral reassortants generated using combinations of CMV genomic RNAs 1, 2 or 3 (subscript numbering) of either Fny-CMV (indicated by F) or LS-CMV (L) were used to inoculate wild-type Arabidopsis plants (Col-0 ecotype). Mock indicates a mock-inoculated plant. Different letters (a-d) are assigned to statistically different results (ANOVA with post-hoc Tukey’s tests, *P*<0.05). Error bars represent standard error of the mean.

**Figure 7:**
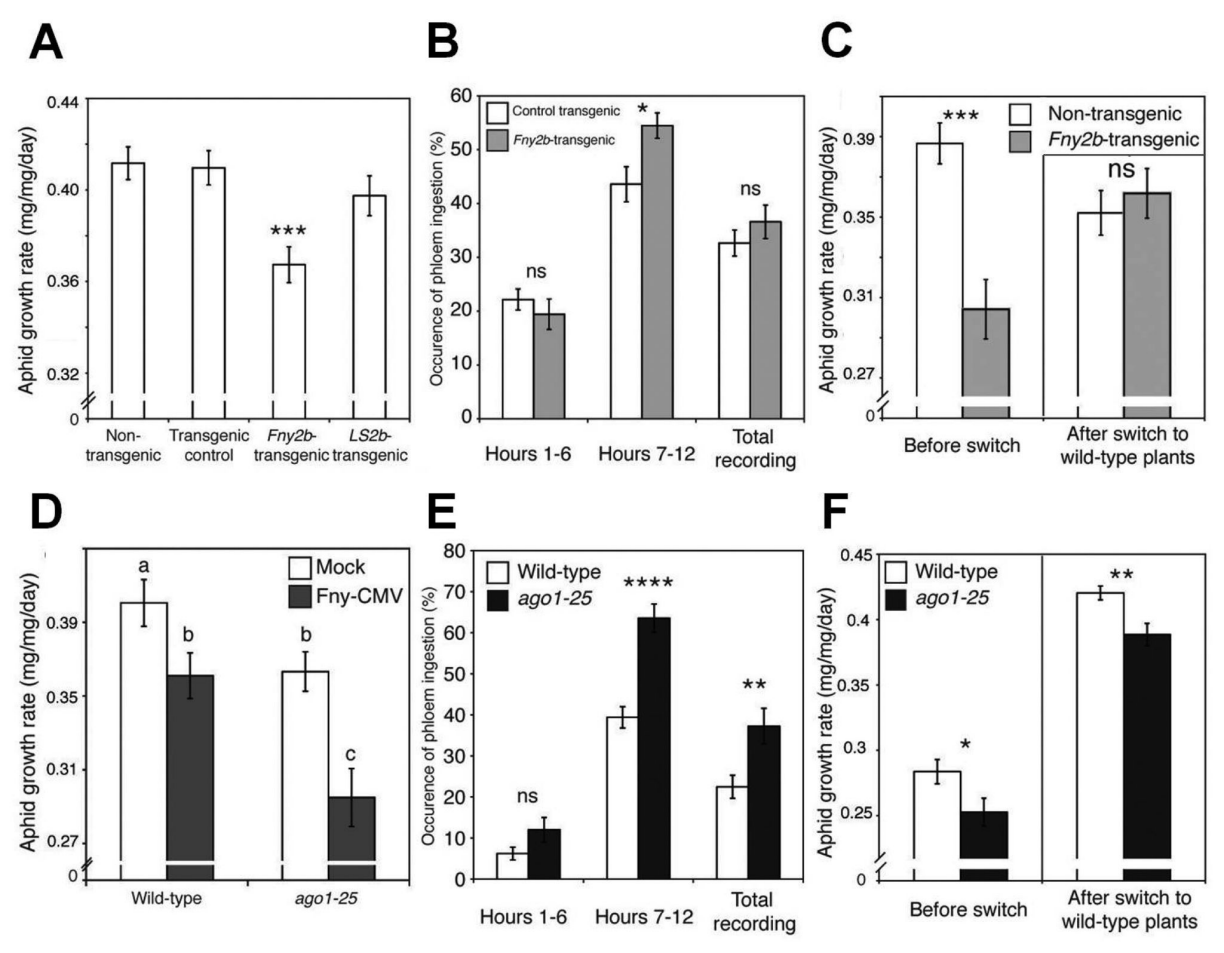
Disruption of AGO1 function by Fny2b induced antibiosis but not feeding deterrence to aphids. (A, D) Growth rate of individual aphids feeding on transgenic plants expressing the Fny2b or LS2b proteins and *ago1-25* mutant plants, which are compromised in AGO1 activity, a major molecular target of Fny2b, n≥24. (B, E) Electrical penetration graph analysis of the amount of phloem feeding in *Fny2b*-transgenic plants and *ago1-25* mutants over a 12-hour recording period, broken down into hours 1-6, 7-12, and over the entire period (C, F) Results of host-switching experiments where aphids were moved from their initial hosts (“before switch”) to uninfected, wild-type plants (“after switch”) to see if aphids could recover from any effects of the initial host on aphid growth rate, n≥24. Results of Student’s *t*-tests are indicated by asterisks: *, *P*<0.05; **, *P*<0.01; ***, *P*<0.001. Error bars represent standard error of the mean.

The CMV genome comprises three positive-sense RNAs. RNA1 encodes protein 1a, RNA2 encodes proteins 2a and 2b, and RNA3 encodes the movement and coat proteins [28]. We found that the strain LS-CMV did not induce aphid resistance in Arabidopsis ecotype Col-0. This made it possible to map the viral inducer of feeding deterrence encoded by Fny-CMV to RNA2 by creating reassortant CMV genomes (see below) containing mixtures of genomic RNAs derived from either Fny-CMV (RNAs indicated by F), or LS-CMV (indicated by L) (Figure 6A). The RNA2-encoded 2a RNA polymerase and the RNA1-encoded 1a methyltransferase/helicase protein are viral replicase components [28,29]. The reassortant F_RNA1(1_)F_RNA2(2_)L_RNA3(3)_ replicated efficiently and induced aphid resistance, whereas F_1_L_2_L_3_ replicated well but did not induce resistance (Figures 6A, 6B, Figure S8). However, efficient CMV replication requires strain-specific 1a-2a compatibility [30], and the combination F_1_L_2_ seemed more compatible than the combination L_1_F_2_. Hence, reassortants L_1_F_2_L_3_ and L_1_F_2_F_3_ accumulated less well (Figure 6B) and did not induce resistance to aphids (Figure 6A), despite possessing Fny-CMV RNA2. The 2b protein is a viral suppressor of RNA silencing (VSR) that is encoded by the 3’ proximal open reading frame (ORF) of RNA2 [28], and was implicated in plant-aphid interactions [9,14]. Therefore, we measured aphid growth rates on transgenic plants constitutively expressing 2b proteins derived from Fny-CMV or LS-CMV [31]. Aphids feeding on *Fny2b*-transgenic but not on *LS2b*-transgenic plants exhibited decreased growth (Figure 7A). 

In Arabidopsis ecotype Col-0, the Fny2b protein, but not the LS2b protein, interferes strongly with micro(mi)RNA-mediated gene regulation. This is thought to be due in part to inhibition of ARGONAUTE1 (AGO1) by the Fny2b protein [31–33]. *M. persicae* growth was inhibited on plants of the mutant lines *ago1-25* and *dcl1-9* (deficient in *DICER-LIKE1*-mediated miRNA biogenesis) (Figure 7D, Figure S9). Thus, AGO1 negatively regulates a miRNA-controlled anti-aphid resistance mechanism(s) (Figure 7D). However, infection of *ago1-25* plants with Fny-CMV intensified aphid resistance, suggesting that the virus can induce additional, AGO1-independent aphid resistance mechanism(s). EPG showed that aphids on *Fny2b*-transgenic and *ago1-25* mutant plants displayed no decrease in phloem ingestion; indeed they showed a marked increase in time spent feeding from phloem on these plants (Figures 7B, 7E, and Figure S10A, S10B). Therefore, aphid resistance in *ago1-25* plants was not explainable by feeding deterrence but was likely due to induction of toxicity; an effect that appears not to have been perceivable by the aphids since they showed no reluctance to feed from the phloem of these plants. Induction of toxicity was confirmed when it was found that aphids placed on *ago1-25* plants were unable to recover normal growth rates following their transfer to wild-type plants (Figures 7C, 7F). Furthermore, accumulation of the feeding-deterrent 4MI3M and other glucosinolates was markedly decreased in *ago1-25* plants (Figure S11). This may explain increased phloem-feeding and suggests AGO1 has regulatory roles that are, respectively, positive for maintaining basal 4MI3M levels, and negative in control of antibiosis (Figure S10B). A role for AGO1 is consistent with previous work on the roles of miRNAs in coordinating defensive secondary metabolism and PTI in Arabidopsis [34,35]. Inhibition of AGO1 activity is less profound in *Fny2b*-transgenic plants than in *ago1-25* mutant plants, and while AGO1-mediated slicing is inhibited by expression of Fny2b, AGO1-mediated translational inhibition is not, meaning that *2b-*transgenic plants incompletely phenocopy *ago1-25* mutants [32,36]. This may explain why aphids reared on *Fny2b*-transgenic plants recovered after transfer to non-transgenic plants, but not after transfer from *ago1-25* plants (Figures 7C, 7F). Although levels of camalexin, a proposed anti-aphid factor [26], were elevated to similar extents in *Fny2b*-transgenic and *ago1-25* plants these levels were lower than those observed after infection of wild-type plants with Fny-CMV (Figures S7A, S7B). Thus, differences in camalexin biosynthesis appear unlikely to explain the toxicity to aphids seen in this system. 

**Figure 8:**
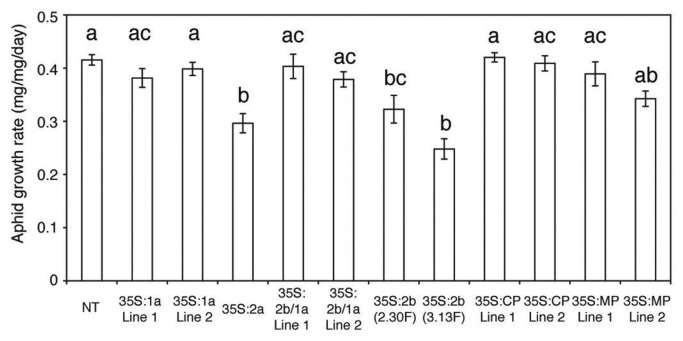
The Fny2a protein induces anti-aphid resistance in transgenic plants. Growth rate of individual aphids feeding on transgenic Arabidopsis plants expressing one or two Fny-CMV proteins and non-transgenic (NT) plants, n≥24. Transgene expression was driven by the constitutive cauliflower mosaic virus 35S promoter. The CMV proteins expressed were the 1a (replicase component), 2a (replicase component), 2b (viral suppressor of RNA silencing), movement protein (MP), and coat protein (CP). Lines indicated by 2b/1a are double transgenic harboring *1a* and *2b* transgenes. The *2b*-transgenic lines 2.30F and 3.13F were constructed by Lewsey et al. [31]. Different letters (a-c) are assigned to statistically different results (ANOVA with post-hoc Tukey’s tests, *P*<0.05). Error bars represent standard error of the mean.

Since aphid resistance in Fny-CMV-infected plants differs mechanistically from resistance in *Fny2b*-transgenic plants we hypothesized that another RNA2-encoded factor triggers 4MI3M accumulation and the feeding deterrence that results from enhanced levels of this chemical. Using transgenic plants expressing different Fny-CMV ORF sequences (Figures S12, S13) we found that *2a*-transgenic and *2b*-transgenic plants were significantly more resistant to aphids than plants of all other lines (Figure 8). Interestingly, *1a/2b* double-transgenic plants showed no increase in aphid resistance (Figure 8), suggesting that the 1a protein antagonizes 2b-induced aphid resistance. Thus, the presence of the 1a protein in infected cells is likely to explain how 2a-induced feeding deterrence predominates over 2b-induced resistance in Fny-CMV-infected plants.

**Figure 9:**
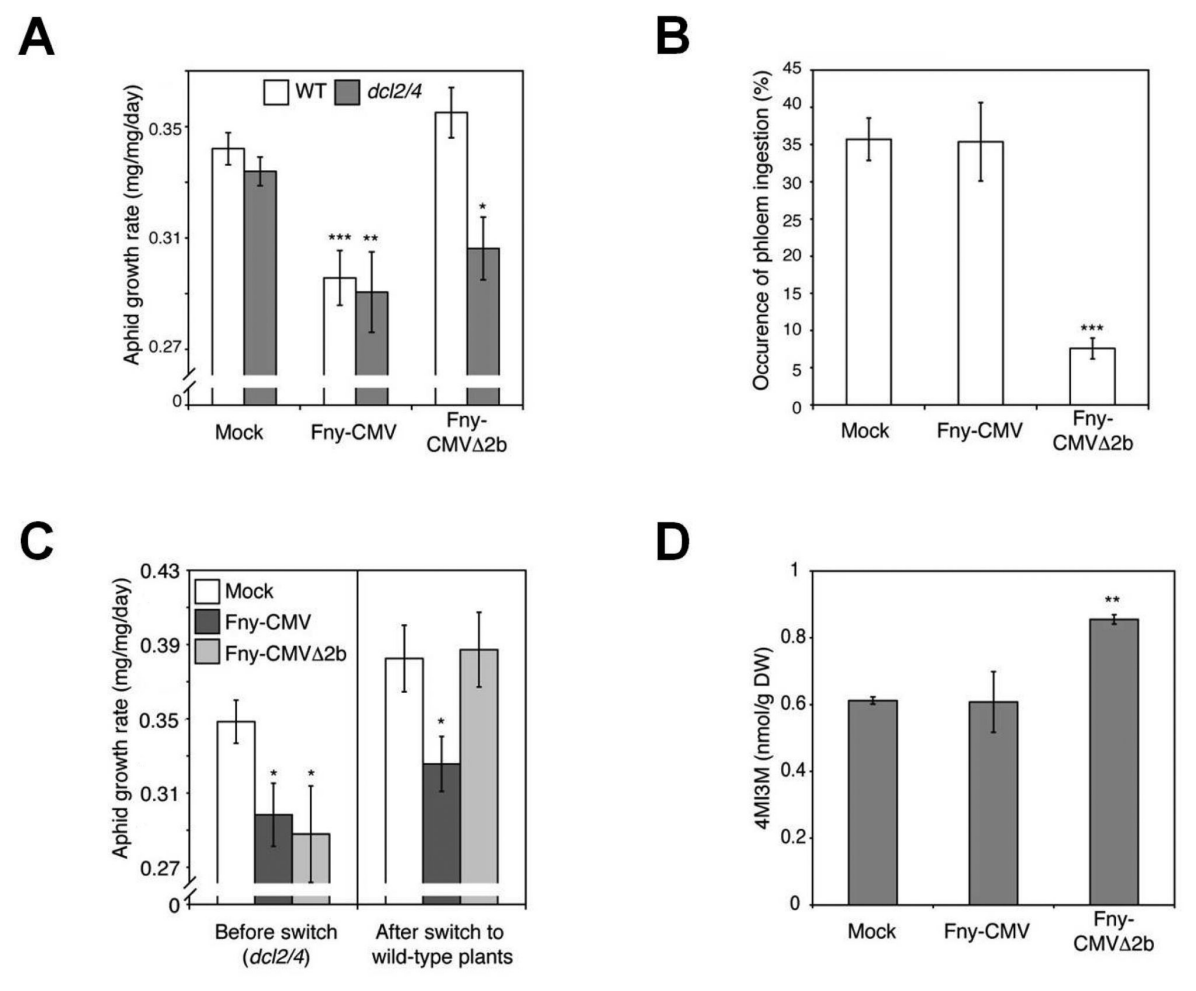
The CMV 2a protein triggers feeding deterrence in CMV-infected Arabidopsis plants. (A) Growth rate of individual aphids placed on *dcl2/4* plants infected with Fny-CMV or Fny-CMVΔ2b, n≥24. (B) Electrical penetration graph analysis of the percentage occurrence of phloem ingestion over 12-hour periods for aphids feeding on *dcl2/4* mutants infected with Fny-CMV or Fny-CMVΔ2b, n=15. (C) Results of host-switching experiments reporting the growth rate of aphids moved from mock-inoculated or virus-infected *dcl2/4* plants (“before switch”) to untouched wild-type plants (“after switch”), n≥24. (D) Accumulation of the aphid feeding deterrent, 4-methoxy-indol3yl-methyl-glucosinolate (4MI3M) in virus-infected *dcl2/4* mutants, measured by high performance liquid chromatography, n=3. Asterisks indicate results of Student’s *t*-tests compared to the mock-inoculated plant of each genotype: *, *P*<0.05; **, *P*<0.01; ***, *P*<0.001. Error bars represent standard error of the mean.

To further distinguish between the effects of the 2a and 2b proteins on aphid resistance in Fny-CMV-infected plants we used the viral mutant, Fny-CMVΔ2b, which cannot express the 2b protein or a portion of the C-terminal domain of the 2a protein due to deletion of nucleotides 2419-2713 of RNA2 [37]. Fny-CMVΔ2b did not affect susceptibility of wild-type Arabidopsis plants to aphids (Figure 9A, Figures S2A, S2B). Since this may be explainable by the lower titers reached by Fny-CMVΔ2b than by Fny-CMV in Arabidopsis (Figure S14) further experiments were done in plants deficient in antiviral silencing (*dcl2/4* double-mutant plants) [38], in which Fny-CMVΔ2b accumulates to levels comparable to wild-type Fny-CMV [39,40] (Figure S14). Aphids confined on Fny-CMV- and Fny-CMVΔ2b-infected *dcl2/4* mutant plants showed decreased growth, which EPG showed was due to feeding deterrence on Fny-CMVΔ2b-infected plants (Figures 9A, 9B, Figure S15). We confirmed that the truncated version of 2a (called here, 2a_Tr) in Fny-CMVΔ2b [37] has the capacity to induce anti-aphid resistance by measuring aphid growth on a *2a*_*Tr*-transgenic line (Figure S13). Aphid growth was significantly reduced on the *2a*_*Tr*-transgenic line (Figure S16). Intriguingly, phloem ingestion was not decreased on *dcl2/4* mutants infected with Fny-CMV, which can produce the 2b protein (Figure 9B, Figure S15). Aphids transferred from Fny-CMV-infected *dcl2/4* mutant plants did not recover normal growth rates (Figure 9C), which is indicative of antibiosis. The 2b protein binds virus-derived siRNAs [41] but in *dcl2/4* mutants, levels of these siRNAs are diminished [38]. Therefore, a higher proportion of 2b protein present in virus-infected plants could bind its other target, AGO1, triggering antibiosis. In host-switching experiments, when aphids were transferred from Fny-CMVΔ2b-infected *dcl2/4* mutant plants to healthy wild-type plants, the insects recovered normal growth rates (Figure 9C), confirming that Fny-CMVΔ2b induced feeding deterrence in *dcl2/4* plants, but not antibiosis. Moreover, in *dcl2/4* Fny-CMVΔ2b, but not Fny-CMV, induced 4MI3M accumulation, although abundance of other glucosinolates was unaffected (Figure 9D, Figure S17). Thus, the 2a protein is most probably the Fny-CMV-encoded factor triggering increased 4MI3M biosynthesis and consequent feeding deterrence.

## Discussion

The induction of feeding deterrence against *M. persicae* in Arabidopsis by infection with Fny-CMV appears to be an emergent property of the direct or indirect interactions of three viral gene products with the host or with each other. During infection, the 2a protein elicited host defense responses that involved signaling components associated with ETI and PTI. This resulted in enhanced biosynthesis of an aphid feeding deterrent, 4MI3M. Experiments with *2b*-transgenic plants showed that the 2b protein has the potential to trigger different form(s) of aphid resistance, most likely by inhibiting AGO1 [32], and based on antibiosis (toxicity) in contrast to antixenosis (feeding deterrence). There are two probable reasons why 2b-induced toxicity was not observed during Fny-CMV infection. Firstly, 2a-induced feeding deterrence may ensure that aphids do not ingest significant amounts of any toxic factor(s) induced by the 2b protein through inhibition of AGO1. Secondly, the 1a protein may moderate the effects of the 2b protein. This idea is supported by the observation that no aphid resistance was apparent in double *1a/2b*-transgenic plants. Cross-talk between the 1a and 2b proteins also appears to inhibit 2b-induced disruption of plant development, which is thought to result from the interaction of the Fny2b protein with AGO1 [31,32]. Thus, although *Fny2b-*transgenic plants phenocopy *ago1-25* mutants and develop deformed leaves [31,32], we found that this did not occur in *1a/2b*-double transgenic plants. A possible explanation for 1a-2b cross-talk comes from Asaoka and colleagues [42] who showed that the cucumoviral 1a protein regulates 2b protein accumulation. Direct or indirect interactions between viral gene products might “tune” host responses to the advantage of the virus. For example, these interactions may modulate 2b-mediated silencing suppression so as to allow binding of virus-derived siRNAs (which will inhibit antiviral silencing), while minimizing inhibition of AGO1 by the 2b protein, which would trigger antibiosis against the virus’ aphid vectors.

**Figure 10:**
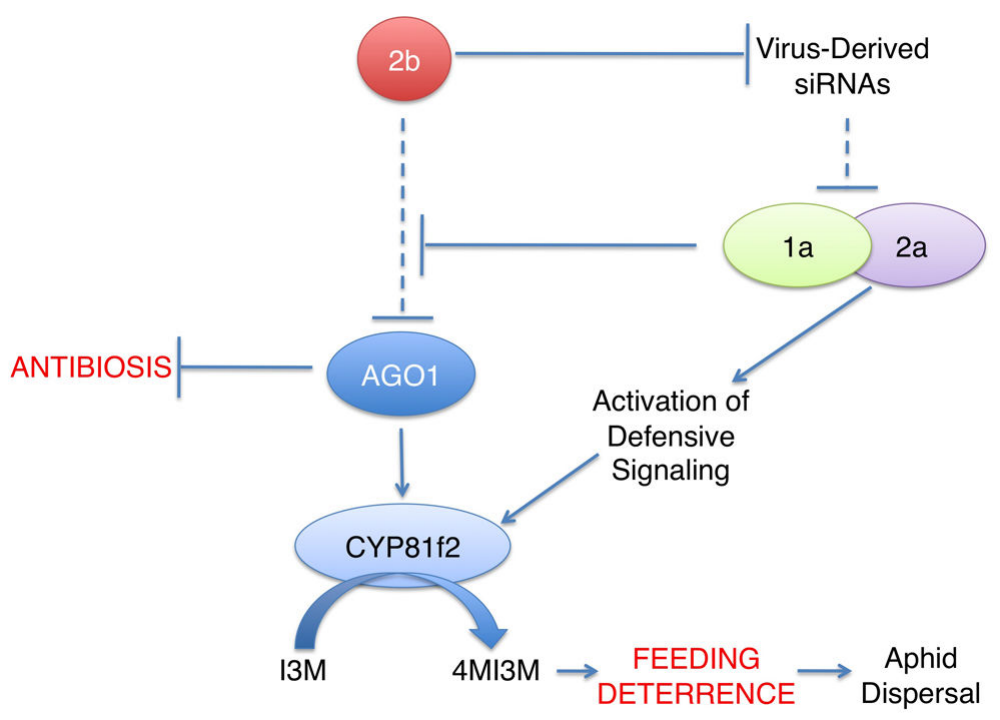
Induction of aphid feeding deterrence and avoidance of aphid antibiosis induction during CMV infection. The 2b RNA silencing suppressor protein of CMV inhibits antiviral silencing through binding of virus-derived siRNAs, allowing viral gene products [41], including the 1a and 2a replicase proteins, to accumulate. The 2b protein can also bind to and inhibit AGO1 [32], which positively regulates expression of the gene for CYP81F2, which catalyzes formation of the aphid feeding deterrent compound 4-methoxy-indol3yl-methylglucosinolate (4MI3M) from its precursor indol-3-yl-methylglucosinolate (I3M). AGO1 also negatively regulates induction of a toxicity-based resistance to aphids (antibiosis). The 1a replicase protein appears to be able to moderate 2b inhibition of AGO1 by the 2b protein, preventing induction of antibiosis and preventing inhibition of 4MI3M biosynthesis. The 2a protein stimulates PTI- and ETI-related signaling, which results in stimulation of *CYP81F2* expression and increased accumulation of 4MI3M. This results in feeding deterrence (see Figure 1A), which is thought likely to increase aphid dispersal and thus enhance transmission of non-persistently aphid-transmitted viruses like CMV (Reviewed in reference [4]). In this diagram dashed lines represent inhibitory processes (siRNA-mediated antiviral silencing and 2b-mediated inhibition of AGO1) that are down regulated during CMV infection.

The VSRs of several viruses target AGO1 as part of their mode of action [43]. For viruses that are not dependent upon aphids for transmission this could be an effective means of inhibiting antiviral silencing. For CMV, however, the 2b-AGO1 interaction could be viewed as a booby trap, since for this aphid-transmitted virus the induction of toxicity to aphids would be prejudicial to successful onward transmission of the virus. Perhaps this is why sequestration of small RNAs, not inhibition of AGO1 activity, is the major means by which the 2b protein suppresses antiviral silencing [33]. Thus, the concerted action of three effectors encoded by CMV allows this virus to overcome RNA silencing-mediated resistance, to avoid triggering strong, toxicity-based resistance against aphids, and to induce synthesis of a feeding deterrent, 4MI3M, through activation of defensive signaling (Figure 10). 

For non-persistently transmitted viruses, brief and shallow probe-feeds with the stylet favor aphid-mediated inoculation and also favor the acquisition of virus particles by these insects. Thus, CMV-induced feeding deterrence, which inhibits prolonged ingestion, is likely to encourage virus transmission [4,44]. However, we observed that there were strain-specific differences in the effects that CMV had on Arabidopsis-aphid interactions. Specifically, Fny-CMV infection induced feeding deterrence but infection with LS-CMV had no discernable effect on the interaction of Arabidopsis with aphids. This might suggest that LS-CMV would not be able to enhance aphid-mediated transmission, at least not between Arabidopsis plants. However, *A. thaliana* is a highly diverse species (discussed in 45), whilst CMV has a very wide host range that encompasses over a thousand plant species [28,46]. So although Arabidopsis and CMV have co-evolved in the wild [11], feeding deterrence may occur only in certain ecotype/CMV strain combinations or during infection of other species within the massive host range of CMV. 

**Figure 11:**
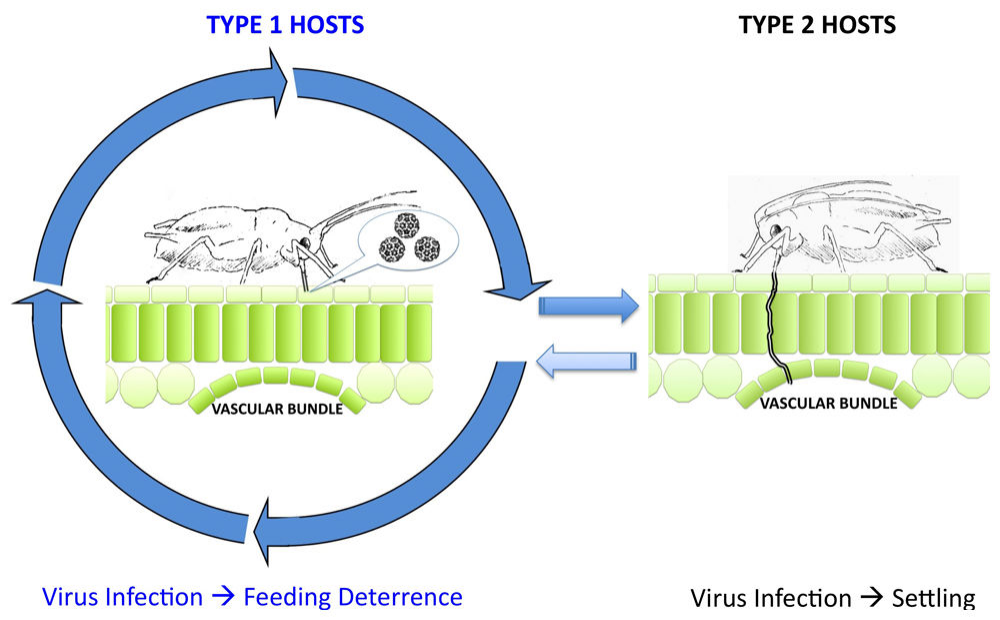
Hypothesis: Differential effects of virus infection on aphid plant interactions may favor either transmission or persistence. CMV infection can engender deterrence to prolonged aphid feeding on certain hosts (Type 1), which discourages settling and provides an incentive to move from host to host (indicated by arrowed circle) (Reviewed in reference [4]). Aphid feeding behavior on Type 1 hosts is predominantly probing of the epidermal cells, which favors the acquisition of virus particles on the stylet (mouthparts) for onward transmission to the next host. On Type 2 hosts CMV infection does not induce resistance to prolonged feeding. Aphids acquire nutrients from the host vascular bundle by deeper probing with their stylets, and are able to settle and reproduce. Migration of aphids (and onward virus transmission) away from Type 2 hosts is less rapid than from Type 1 hosts but Type 2 hosts provide an environment that allows aphid populations to recover and to survive challenging conditions (for example drought, cold).

The ecotype- or species-specific effects of CMV and its gene products on plant-aphid interactions will depend in part upon the characteristics of secondary metabolism in the host. Antibiotic (toxic) and antixenotic (anti-feedant) secondary metabolites vary greatly between plant species and the signaling mechanisms controlling secondary metabolism are not well understood in plants other than Arabidopsis. In Arabidopsis we have seen that transgenic expression of the 2b protein from Fny-CMV induced antibiosis but that during viral infection or in double *1a/2b*-transgenic plants the 1a protein countered 2b-induced antibiosis. Perhaps something similar is occurring in squash infected with Fny-CMV, in which feeding deterrence was also induced [8]. However, the secondary metabolism of cucurbits is unlike that of Arabidopsis, so the biochemical ‘outputs’ of viral manipulation of defensive signaling in squash cannot be the same. Contrastingly, in tobacco the 2b VSR *inhibited* the induction of antibiosis by one or more of the other gene products of Fny-CMV and infection with this virus enhanced aphid survival and reproduction [9]. Hence, depending upon the host and virus strain involved, CMV infection can generate one of two extended phenotypes; that is, two distinct types of alteration in host characteristics resulting from the action of parasite (in this instance virus) genes on the host [47]. But what would be the advantage to the virus? We hypothesize that this pleiotropy may suit the needs of the virus and its aphid vectors to adapt to seasonal changes or changes in host availability over time. Plants in which virus infection engenders feeding deterrence (Type 1 hosts: Figure 11) will deter prolonged settling by aphids but promote onward transmission of the virus. However, the reproduction and survival of aphids on these plants is compromised if they are confined or if this is the only plant type available, as shown in this study (Figure 1B). Thus, if one imagines a situation in which only Type 1 hosts were available this could eventually lead to local extinction of the vector, leaving the virus with no means of transmission. However, plants in which viruses inhibit aphid resistance (or at least induce no deleterious effects on the aphids) will foster aphid survival and reproduction (Type 2 hosts: Figure 11). Although onward transmission of the virus from Type 2 hosts will be less frequent, and probably driven by overcrowding of the aphids as their populations grow, these plants may provide a safe haven for aphids during stressful periods of cold or drought [48,49] and allow the vector population to recover after a period of active transmission between Type 1 plants. When conditions improve Type 2 hosts could act as centers from which the aphids and the virus will spread to Type 1 plants and resume a more active phase of transmission. Thus, Type 1 hosts favor rapid transmission of the virus, while Type 2 hosts favor the longer-term persistence of the virus and its vector within a plant community.

Aphid-vectored viruses pose an increasing problem for many major crops. This is likely due to increased insecticide resistance among aphids and because of climatic change affecting the geographic ranges of these insects and the viruses that they transmit [3]. In the future, improved understanding of how viruses influence aphid interactions with crop plants and with the surrounding wild plant population could be used to inform strategies aimed at inhibiting virus transmission and/or the persistence of viruses and their aphid vectors in the agricultural environment. 

## Materials and Methods

### Plants, Aphids and Viruses

The *2b*-transgenic lines (background: *Arabidopsis thaliana* (L.) Heynh. ecotype Col-0) used in this study were 3.13F (Fny2b) and UNT (a line constitutively expressing an untranslatable *Fny2b* transcript), which were described by Lewsey et al. [31]. Seeds for *ago1-25* [50], double mutant *dcl2/4* [38], and *cyp81f2* [23] were from pools previously authenticated for mutant allele studies [23,39,51]. Since homozygous *dcl1-9* mutants [52,53] are sterile, homozygotes were selected from a segregating population by kanamycin resistance, stunted phenotype and (following experimentation) authentication by PCR with allele-specific primers. 

Aphids of *Myzus persicae* (Sulzer) clone US1L [54] were maintained on *Brassica pekinensis* plants. To obtain aphids of standardized developmental stage for use in experiments, adults were transferred to uninfested *B. pekinensis* and allowed to reproduce for no longer than 24 hours. Nymphs produced were transferred to experimental plants using fine paintbrushes and contained using microperforated plastic bags (Associated Packaging, Kent, UK). 

Fny-CMV [55,56], LS-CMV [57], and Fny-CMVΔ2b [37,58] were propagated in tobacco (*Nicotiana tabacum* L. cv. Xanthi-nc) or *N. clevelandii* and virions purified according to Ng and Perry [59]. Virions (100 µg.ml^-1^ in sterile water) were rub-inoculated onto Carborundum-dusted leaves of Arabidopsis plants at the four- to six-true-leaf stage [14]. Mock inoculation used water only. Infection was confirmed by symptom observation and double antibody sandwich ELISA kits (Bioreba, Reinach, Switzerland). 

### Construction of reassortant viruses

CMV reassortants were constituted according to the methods of Zhang et al. [60]. Full-length cDNA clones of Fny-CMV (pF109, pF209, and pF309) and LS-CMV (pLS109, pCL2b-WT-2, and pLS309) were linearised and polished using the Klenow fragment of *E. coli* DNA polymerase I (Fermentas, St Leon-Rot, Germany). cDNA-derived infectious RNAs were generated by *in vitro* transcription with T7 polymerase and the mMessage mMachine kit (Ambion). Resultant RNA transcripts were mixed in various combination to reconstitute either the wild-type viruses, F_1_F_2_F_3_ (Fny-CMV) and L_1_L_2_L_3_ (LS-CMV), or to create the reassortant forms: F_1_L_2_L_3_; L_1_F_2_L_3_; L_1_L_2_F_3_; L_1_L_2_L_3_; F_1_F_2_L_3_; F_1_L_2_F_3_, and L_1_F_2_F_3_. These were inoculated onto plants as described above and infection confirmed by ELISA.

### Generation of transgenic Arabidopsis expressing CMV proteins

DNA sequences containing ORFs encoding the 1a (replication) protein, movement protein (3a) and coat protein (CP) of CMV were amplified by PCR using the proof-reading Pfu polymerase (Finnzymes) from the plasmids pT149 (gift from Dr Tomas Canto) and pFny309 [61] using primers containing appropriate restriction sites (Table S1) that aided subcloning into the plant transformation vectors pBI121 [62] or pDJSn [63] from which the *AOX* cDNA sequence had been removed. This placed the virus-derived sequences under control of the cauliflower mosaic virus 35S promoter and allowed kanamycin or hygromycin selection for pBI121 or pDJSn derived constructs, respectively, *in planta*. Sequences containing the ORFs encoding the 2a replication protein and the truncated 2a protein were amplified by PCR from pFny209 [61] and pF209Δ2b [37] and cloned into the Gateway entry vectors pDONR201 and pDONR207 (Invitrogen) to allow their subsequent mobilization by recombination into vectors pLX222 and pMDC32. This placed them under the control of the cauliflower mosaic virus 35S promoter and allowed kanamycin or hygromycin selection for pLX222- [64] or pMDC32-derived [65] constructs respectively *in planta*. The recombinant plasmids were electroporated into *Agrobacterium tumefaciens* (GV3101). Arabidopsis ecotype Col-0 and plants of the *Fny2b*-transgenic line 2.30F [31] were super-transformed by floral dipping [66] and at least two independent lines resulting from each transformation were used in subsequent experiments.

### Verification of protein expression in the transgenic Arabidopsis plants

Readily detectable levels of CP were evident in the *CP*-transgenic Arabidopsis plants using DAS-ELISA. As antibody signals can be weak for other CMV proteins expressed *in planta* [67,68] the ability of the *1a* and *2a* transgenes to complement replication of combinations of either RNA 2 and 3 or RNA 1 and 3, respectively, was assayed by DAS-ELISA for the presence of CP in the upper, non-inoculated leaves of the transgenic plants. This approach demonstrated that transgenic expression of the 1a and 2a proteins supported systemic infection in plants inoculated with either a mixture of synthetic Fny-CMV RNAs 2 and 3 (transcribed *in vitro* from pFny209 and pFny309, respectively) or a mixture of synthetic RNAs 1 and 3 (transcribed *in vitro* from pFny109 and pFny309, respectively) indicating that functionally and/or enzymatically active proteins were being expressed (Figure S13). Transgenic expression of biologically active MP also complemented the systemic spread of CMV after inoculation with RNA 1 and 2 with a modified RNA 3 encoding the green fluorescent protein (GFP) gene in place of the *MP* ORF transcribed from the plasmid pF:GFP/CP as described previously [58,69]. 

### Aphid performance

Mean relative growth rate (MRGR) of aphids was calculated using the formula MRGR = (log_*e*_ *W*_*final*_ - log_*e*_ *W*_*initial*_)/*t*, where *t* = time (days) between initial and final measurements of aphid fresh weight (*W*) [70,71]. One-day-old first instar nymphs were individually weighed on a microbalance (Mettler/Toledo Model MX5) before being placed on test plants. The final weight of each aphid was measured five days post-infestation. At least 24 replicates per treatment group were used and experiments performed at least twice unless otherwise stated. Aphid colony size was measured by counting the total number of aphids produced from an initial placement of a single one-day-old first instar nymph at 10 days post-infestation.

### EPG techniques

The direct-current EPG method [12] was used to monitor aphids’ feeding activity on Arabidopsis leaves. Individual apterous adult aphids were pre-starved for 30-60 mins and attached to a 4 cm tether of 20µm diameter gold wire (EPG systems, Wageningen, The Netherlands) using conductive silver paint (Electrolube, Swadlincote, UK, or EPG systems). The gold wire was soldered to a 1 cm brass pin, connected to an amplifier with 1 GΩ resistance and 50-100X gain. Connected aphids were placed on individual plants inside a Faraday cage and signals received from the EPG monitor were displayed and analyzed using PROBE 3.4 software (EPG systems). Waveforms were scored according to Tjallingii and Hogen Esch [72]. Relevant aphid behavior-related EPG parameters were calculated using Microsoft Excel-based spreadsheets developed by Dr Edgar Schliephake (Julius Kuhn Institute, Germany) and by Sarria et al. [73].

### Microarray and RT-Q-PCR analysis of plant gene expression

Aerial tissues were harvested at 14 days post-inoculation from Fny-CMV-infected, Fny-CMVΔ2b-infected or mock-inoculated plants. Total RNA was extracted using TRIzol reagent (Invitrogen, Carlsbad, CA, USA) following the methods of Lewsey et al. [14]. Three independent biological replicates were performed. Array hybridization and scanning was conducted by the Nottingham Arabidopsis Stock Centre Affymetrix Service (Experiment reference: NASCARRAYS-552 "The effects of infection with wild-type or mutant cucumber mosaic virus on the Arabidopsis transcriptome"). Raw data of probe sets were quantile-normalized using the MAS5 algorithm method [74]. Probe sets with signal intensity values above the 20th percentile of all signal intensity values were used to analyze significantly expressed genes. For each gene *P* values were calculated using paired *t*-tests. Significantly differentially regulated genes were determined on the threshold of *P*<0.05 and fold change ≥2.0. Corrections were made to account for multiple comparisons using the Benjamini and Hochberg False Discovery Rate test with GeneSpring software (Agilent Technologies). However, this proved to be too conservative to detect significant changes in gene expression, so data were further analyzed without correction.

To validate microarray results several transcripts were selected for RT-Q-PCR analysis. First-strand synthesis was carried using SuperScript III (Invitrogen) reverse transcriptase primed using random hexamers (Promega, Madison, WI, USA) and quantitative PCR was conducted using SYBR Green JumpStart *Taq* ReadyMix (Sigma-Aldrich, St Louis, MO, USA) using primers specific to the transcript of interest selected from Table S2. Expression levels for reference gene (*ELONGATION* FACTOR *1α* or *GAPDH*) were stable under the conditions used. Reactions were conducted in triplicate. Data were analyzed using LinRegPCR software to calculate threshold cycle number and amplification efficiency [75,76]. Fold-changes in transcript abundance were calculated using ΔΔCt methodology, adjusted for amplicon amplification efficiency taking into account the reference transcript to control for variations in loading [77], and expressed relative to transcript abundance in mock-inoculated wild-type plants.

### Secondary metabolite extraction and analysis

For analysis of glucosinolates approximately 20 mg freeze-dried tissue was extracted under methanol according to Rossiter et al. [78]. Samples were centrifuged and supernatants purified on DEAE-A25 ion exchange columns (Bio-Rad). Sulfatase was added to each column and incubated overnight before glucosinolates were eluted with water and concentrated by freeze-drying. Samples were resuspended in 200 μl water and analyzed by reverse-phase HPLC on a C18 column (Phenomenex, Torrance CA, USA). Glucosinolate concentrations were calculated using response factors documented in Brown et al. [79], and adjusted according to the recovery rate of an internal standard (sinigrin).

To examine aphid-induced camalexin accumulation, thirty *M. persicae* nymphs were confined on single leaves of intact five-week-old Arabidopsis plants with clip-cages following the methods described by Kettles et al. [26]. Leaves of control plants had empty clip-cages placed upon them. Both mock and aphid-infested leaves were harvested after 48 hours. Camalexin was extracted from leaves of infested and non-infested plants and analyzed by reverse-phase HPLC and fluorescence detection as previously described for SA analysis [80] but with modified excitation and emission wavelengths optimized for camalexin [27]. Camalexin was quantified by comparison with a standard curve obtained by using pure, synthetic camalexin (generous gift from Professor Jane Glazebrook, University of Minnesota).

### GUS histochemical assay

Arabidopsis rosettes were vacuum infiltrated for 15 minutes with GUS substrate solution [100 mM sodium phosphate, pH 7.0, 0.5 mM K_3_Fe(CN)_6_, 0.5 mM K_4_Fe(CN)_6_, 10 mM EDTA, 0.01% Tween 20, 1M 5-bromo-4-chloro-3-indolyl-β-D-glucuroni​c acid] and incubated at 37°C overnight. Tissue was washed several times in 70% ethanol before observation of the indigo staining [15].

### Statistics

Minitab v15 (Minitab Ltd, Coventry, UK) was used for all statistical analyses and tests. Datasets were checked for normality using an Anderson-Darling test [81]. Datasets that did not follow a normal distribution were transformed using the Johnson method [82,83]. Normalized data was analyzed using General Linear Model ANOVA and subsequent comparisons conducted using *post-hoc* Tukey’s tests. Frequency data was analyzed using the χ-squared test of independence.

## Supporting Information

Figure S1 **Aphid migration behavior is enhanced by CMV infection of wild-type Arabidopsis plants.** Ten aphids were released onto rosettes of mock-inoculated or Fny-CMV-infected release plants and then allowed to remain or emigrate to a plant of the opposite treatment group located 10 cm away in the same pot. Aphids migrated away more often from Fny-CMV-infected than from mock-inoculated plants. Fny-CMV infected choice plants arrested fewer aphids at 24 hours relative to mock-inoculated plants. Based on the methods of Mauck et al. [8], three independent tests were performed for each type of release plant. See Figure 1A for the accompanying aphid migration data. Error bars represent standard error of the mean. Asterisks indicate significant differences (Student’s *t*-test): *, *P*<0.05; **, *P*<0.01; ***, *P*<0.001.
(TIF)

Figure S2 **Aphid behavior and performance on virus-infected wild-type plants.** (A) Production of aphid nymphs from an initial infestation of two one-day-old nymphs (per plant) was monitored over a 10 day period, n=6. Statistically significant (ANOVA with *post-hoc* Tukey’s tests) differences are indicated: *, *P*<0.05, and **, *P*<0.001. (B) Aphid colony size produced from initial infestations of single one-day-old nymph at 14 days post-infestation, n≥10. Different letters are assigned to significantly different groups (ANOVA with *post-hoc* Tukey’s tests, *P*<0.05). Error bars represent standard error of the mean.
(TIF)

Figure S3 **Percentage occurrence of waveforms produced in electrical penetration graph (EPG) analysis of feeding aphids over 12-hour recordings.** Ingestion from the phloem (E2 waveform; black) was significantly decreased for aphids feeding on Fny-CMV-infected plants. Statistical significance was tested by Student’s *t*-test compared to percentage occurrence of phloem ingestion on mock-inoculated plants for the first and second halves of the recording and for the whole recording.
(TIF)

Figure S4 **Confirmation of the responses of selected salicylic acid- and jasmonic acid-regulated transcripts to Fny-CMV and Fny-CMVΔ2b infection by RT-Q-PCR at 14 days post-inoculation.** Mean fold change in expression was calculated relative to the expression of each gene in mock-inoculated plants. Error bars represent standard error of the mean.
(TIF)

Figure S5 **Glucosinolate accumulation in virus-infected wild-type plants.** (A-D) Glucosinolate accumulation in Fny-CMV- and Fny-CMVΔ2b-infected wild type plants was analyzed using reverse phase high performance liquid chromatography. Error bars represent standard error of the mean. Abbreviation: I3M, indol-3-yl-methylglucosinolate. Different letters are assigned to significantly different results (ANOVA with *post-hoc* Tukey’s tests *P*<0.05).
(TIF)

Figure S6 **Aphid performance on virus-infected wild-type and *pmr4-1* mutant plants.** Mean relative growth rate of individual aphids feeding on *pmr4-1* mutant (which cannot accumulate callose) and wild type (WT) plants, n≥24. Error bars represent standard error of the mean. Different letters are assigned to significantly different groups (ANOVA with *post-hoc* Tukey’s tests, *P*<0.05).
(TIF)

Figure S7 **Camalexin does not play a major role in Fny-CMV-induced resistance to *Mysus persicae* in Arabidopsis.** (A) Differences in camalexin accumulation in Arabidopsis leaves 48 hours post-infestation with 30 aphid nymphs were small compared to leaves systemically infected with Fny-CMV or Fny-CMVΔ2b, n=3. Statistically significant (ANOVA with *post-hoc* Tukey’s tests) differences are indicated: **, *P*<0.001. See panel (B) for statistics on the aphid-infested plants. (B) Detailed examination of camalexin accumulation in aphid-infested leaves [also shown in panel (A)] revealed that in plants with disrupted miRNA utilization (*35S:2b*-expressing lines 2.30F and 3.13F and *ago1-25*), basal levels of camalexin were significantly higher (ANOVA with *post-hoc* Tukey’s tests, *P*<0.01) than in non-transformed (NT) plants and *pad3-1* mutant plants. Interestingly, the double transgenic plants expressing the 2b and 1a proteins had levels of camalexin similar to NT plants. Aphid infestation resulted in significantly increased levels of camalexin relative to the mock-treatment in the *35S:2b*-expressing line 2.30F and in the mutant *ago1-25* (ANOVA with *post-hoc* Tukey’s tests, *P*<0.05). (C) The mean relative growth rate of individual aphids feeding on Fny-CMV-infected wild-type and *pad3-1* mutants (impaired in camalexin biosynthesis) was lower compared to aphids feeding on mock-inoculated plants, n≥24. The decrease in aphid growth rate on Fny-CMV-infected *pad3-1* mutants was slightly less severe than in virus-infected wild-type plants (ANOVA with *post-hoc* Tukey’s tests, *P*<0.05) indicating that camalexin can make only a minor contribution at best to aphid resistance induced by Fny-CMV. Error bars represent standard error of the mean. Different letters are assigned to significantly different groups.
(TIF)

Figure S8 **Symptoms induced by CMV reassortants in wild-type Arabidopsis plants.** Viral reassortants generated using combinations of CMV genomic RNAs 1, 2 or 3 (subscript) from either Fny-CMV (F) or LS-CMV (L) were used to inoculate wild-type Arabidopsis plants (Col-0 ecotype). Mock indicates a mock-inoculated plant. Plants were photographed at 14 days post-inoculation. Scale bars represent 1 cm.
(TIF)

Figure S9 **Aphid performance on virus-infected wild-type plants and *dcl1-9* mutant plants.** Mean relative growth rate of individual aphids feeding on *dcl1-9* mutant and wild type (WT) Arabidopsis plants, n≥24. Error bars represent standard error of the mean. Different letters are assigned to significantly different groups (ANOVA with *post-hoc* Tukey’s tests, *P*<0.05).
(TIF)

Figure S10 **Percentage occurrence of waveforms produced in electrical penetration graph (EPG) analysis of feeding aphids over 12-hour recordings.** (A) Ingestion from the phloem (E2 waveform, colored black) was significantly increased for aphids feeding on *Fny2b*-transgenic plants in the second half of recordings. (B) Ingestion from the phloem (E2 waveform, colored black) was significantly increased for aphids feeding on *ago1-25* transgenic plants across the whole recording. Statistical significance was tested by Student’s *t*-test compared to percentage occurrence of phloem ingestion on mock-inoculated plants for the first and second halves of the recording and for the whole recording.
(TIF)

Figure S11 **Glucosinolate accumulation in wild-type plants and *ago1-25* mutants.** Glucosinolate accumulation of Fny-CMV-infected wild-type plants and *ago1-25* mutants was analyzed using high performance liquid chromatography, n=3. Error bars represent standard error of the mean. Abbreviations: 4MI3M, 4-methoxy-indol3yl-methylglucosinolate, and I3M, indol-3-yl-methylglucosinolate. Different letters are assigned to significantly different results (ANOVA with *post-hoc* Tukey’s tests *P*<0.05).
(TIF)

Figure S12 **Phenotypes of transgenic Arabidopsis plants constitutively expressing various Fny-CMV proteins.** Appearance of plants (from independent transformed lines) expressing transgenes encoding the CMV proteins 1a, 2a, 2b, movement protein (MP), and coat protein (CP) under the control of the constitutive cauliflower mosaic virus 35S promoter. Vector control is a plant from a line transformed with ‘empty’ pBI121.1. Plants were five weeks old when photographed. The *2b*-transgenic lines 2.30F and 3.13F were constructed by Lewsey and colleagues [31]. Scale bar represents 1 cm.
(TIF)

Figure S13 **CMV proteins transgenically expressed in Arabidopsis are biologically active.** (A) The transgenic plants expressing the *1a* [1a (c)] or *2b* and *1a* [2b/1a (a) and 2b/1a (b)] ORFs of Fny-CMV were inoculated with a mixture of synthetic RNAs 2 and 3 (RNA 2+3) generated by *in* *vitro* transcription of the plasmids pFny209 and pFny309, respectively. Three weeks post-inoculation, CMV coat protein (CP) was detected by DAS-ELISA in the non-inoculated leaves indicating that virus replication had occurred (due to complementation of efficient replication and spread by the transgenically-expressed viral protein). (B) Transgenic plants expressing the *2a* (2a) and truncated *2a* (2a_Tr) ORFs were inoculated with synthetic RNAs 1 and 3 (RNA 1+3) generated by *in* *vitro* transcription of the clones pFny109 and pFny309, respectively. Three weeks post inoculation CMV CP was detected by DAS-ELISA in the non-inoculated leaves indicating that virus replication had occurred and therefore that the *2a*-derived transgenes were expressing enzymatically active 2a (RNA-dependent RNA polymerase) protein and complementing efficient viral replication and spread. (C) Transgenic plants expressing the *MP* gene [independent lines MP (a) and MP (c)] were inoculated with RNA 1 and 2 and a modified RNA 3 containing the *GFP* gene in place of the *MP* ORF (RNA 1+2+CP), where CP represents the synthetic RNA transcribed from pF:GFP/CP as described previously (58,69). Three weeks post-inoculation, CMV CP was detected by DAS-ELISA in the non-inoculated leaves indicating that the transgenically expressed MP had complemented cell-to-cell movement and facilitated systemic movement of RNAs 1, 2 and the modified RNA 3. (D) Expression of CP was directly detected by DAS-ELISA in leaf tissue from transgenic plants expressing the *CP* ORF. Additional transgenic plants in (A-C) have been inoculated with a mixture of RNAs 1, 2, and 3 (RNA1+2+3), which constitutes the whole Fny-CMV genome, as a positive control. Non-transgenic (NT) plants and mock-inoculated (Mock) transgenic plants gave a background signal in DAS-ELISA.
(TIF)

Figure S14 **Relative accumulation of Fny-CMV and Fny-CMVΔ2b in wild-type and *dcl2/4* double mutant plants.** Viral RNA accumulation was measured (by RT-Q-PCR) relative to the accumulation of Fny-CMV RNA in wild-type plants at 14 days post-inoculation, n=3. Error bars represent standard error of the mean. the experiments was repeated three times with similar results. Different letters are assigned to statistically different groups (ANOVA with *post-hoc* Tukey’s tests, *P*<0.05).
(TIF)

Figure S15 **Aphid feeding behavior on *dcl2/4* mutant plants: Percentage occurrence of waveforms produced in electrical penetration graph (EPG) analysis of feeding aphids over 12-hour recordings on virus infected *dcl2/4* mutants.** Ingestion from the phloem (E2 waveform, colored black) was significantly increased in the second half of the recording for aphids feeding on Fny-CMV infected *dcl2/4* mutant plants. Ingestion from the phloem was significantly decreased for aphids feeding on Fny-CMVΔ2b-infected *dcl2/4* mutant plants. Statistical significance was tested by Student’s *t*-test compared to percentage occurrence of phloem ingestion on mock-inoculated plants for the first and second halves of the recording and for the whole recording.
(TIF)

Figure S16 **A truncated form of the Fny2a protein had the capacity to induce anti-aphid resistance.** Growth rate of individual aphids feeding on transgenic plants expressing a truncated form of the Fny2a protein (35S:2a_Tr) and non-transgenic (NT) plants, n≥24. Error bars represent standard error of the mean. Asterisks indicate significant differences (Student’s *t*-test): *, *P*<0.05; **, *P*<0.01; ***, *P*<0.001.
(TIF)

Figure S17 **High performance liquid chromatography analysis of glucosinolate accumulation in Fny-CMV-infected (Fny-CMV) and Fny-CMVΔ2b-infected (Fny-CMVΔ2b) *dcl2/4* double mutant plants.** Statistical analysis did not reveal any significant differences compared to mock-inoculated plants (Mock) (ANOVA with *post-hoc* Tukey’s tests, *P*>0.05), except for the increased accumulation of 4-methoxy-indol-3-yl-methylglucosinolate in Fny-CMVΔ2b-infected *dcl2/4* double mutants (see Figure 9D of the main text). Error bars represent standard error of the mean. Data presented represents the mean accumulation of each glucosinolate extracted from tissue from at least three plants per treatment grouped and repeated independently three times.
(TIF)

Spreadsheet S1 (XLS)

Spreadsheet S2 (XLS)

Spreadsheet S3 (XLS)

Table S1 **Primers used in generation of transgenic plants expressing proteins derived from Fny-CMV.**
(DOC)

Table S2 **Primers used in RT-Q-PCR analyses.** *ELONGATION* FACTOR *1α* (EF1*α*) and *GAPDH* were used as stable reference transcripts to control for loading.
(DOC)

## References

[1] Handford MG, Carr JP (2006) Plant metabolism associated with resistance and susceptibility. In: Loebenstein G Carr JP Natural resistance mechanisms of plants to viruses. Berlin: Springer pp. 315-340.

[2] Ng JCK, Perry KL (2004) Transmission of plant viruses by aphid vectors. Mol Plant Pathol 5: 505-511. doi:10.1111/j.1364-3703.2004.00240.x. PubMed: 20565624. 20565624

[3] Westwood JH, Stevens M (2010) Resistance to aphid vectors of virus disease. Adv Virus Res 76: 179-210. PubMed: 20965074. 20965074 10.1016/S0065-3527(10)76005-X

[4] Mauck KE, Bosque-Perez NA, Eigenbrode SD, De Moraes CM, Mescher MC (2012) Transmission mechanisms shape pathogen effects on host-vector interactions: evidence from plant viruses. Functional Ecol 26: 1162-1175. doi:10.1111/j.1365-2435.2012.02026.x.

[5] Blanc S, Uzest M, Drucker M (2011) New research horizons in vector-transmission of plant viruses. Curr Opin Microbiol 14: 483-491. doi:10.1016/j.mib.2011.07.008. PubMed: 21788152. 21788152

[6] Powell G (2005) Intracellular salivation is the aphid activity associated with inoculation of non-persistently transmitted viruses. J Gen Virol 86: 469-472. doi:10.1099/vir.0.80632-0. PubMed: 15659767. 15659767

[7] Boquel S, Giordanengo P, Ameline A (2011) Divergent effects of PVY-infected potato plant on aphids. Eur J Plant Pathol 129: 507-510. doi:10.1007/s10658-010-9732-8.

[8] Mauck KE, De Moraes CM, Mescher MC (2010) Deceptive chemical signals induced by a plant virus attract insect vectors to inferior hosts. Proc Natl Acad Sci U S A 107: 3600-3605. doi:10.1073/pnas.0907191107. PubMed: 20133719. 20133719 PMC2840436

[9] Ziebell H, Murphy AM, Groen SC, Tungadi T, Westwood JH et al. (2011) Cucumber mosaic virus and its 2b RNA silencing suppressor modify plant-aphid interactions in tobacco. Sci Rep 1: 187 PubMed: 22355702. 22355702 10.1038/srep00187 PMC3240964

[10] Zaitlin M, Anderson JM, Perry KL, Zhang L, Palukaitis P (1994) Specificity of replicase-mediated resistance to cucumber mosaic virus. Virology 201: 200-205. doi:10.1006/viro.1994.1286. PubMed: 8184532. 8184532

[11] Págan I, Fraile A, Fernandez-Fueyo E, Montes N, Alonso-Blanco C et al. (2010) *Arabidopsis* *thaliana* as a model for the study of plant-virus co-evolution. Philosophical Trans Royal Soc of London B-Biological Sciences 365: 1983-1995. doi:10.1098/rstb.2010.0062. PMC2880114 20478893

[12] Tjallingii WF (1978) Electronic recording of penetration behaviour by aphids. Entomologia Experimentalis et Applicata 24: 721-730. doi:10.1111/j.1570-7458.1978.tb02836.x.

[13] Whitham SA, Quan S, Chang HS, Cooper B, Estes B et al. (2003) Diverse RNA viruses elicit the expression of common sets of genes in susceptible *Arabidopsis* *thaliana* plants. Plant J 33: 271-283. doi:10.1046/j.1365-313X.2003.01625.x. PubMed: 12535341. 12535341

[14] Lewsey MG, Murphy AM, MacLean D, Dalchau N, Westwood JH et al. (2010) Disruption of two defensive signaling pathways by a viral RNA silencing suppressor. Mol Plant Microbe Interact 23: 835-845. doi:10.1094/MPMI-23-7-0835. PubMed: 20521947. 20521947

[15] Hunter LJR, Westwood JH, Heath G, Macaulay K, Smith AG et al. (2013) Regulation of *RNA-dependent* *RNA* *polymerase* *1* and *Isochorismate* *Synthase* gene expression in Arabidopsis. PLOS ONE 8: e66530. doi:10.1371/journal.pone.0066530. PubMed: 23799112. 23799112 PMC3684572

[16] Zarate SI, Kempema LA, Walling LL (2007) Silverleaf whitefly induces salicylic acid defenses and suppresses effectual jasmonic acid defenses. Plant Physiol 143: 866-875. PubMed: 17189328. 17189328 10.1104/pp.106.090035 PMC1803729

[17] Asai T, Tena G, Plotnikova J, Willmann MR, Chiu WL et al. (2002) MAP kinase signalling cascade in *Arabidopsis* innate immunity. Nature 415: 977-983. doi:10.1038/415977a. PubMed: 11875555. 11875555

[18] Boudsocq M, Willmann MR, McCormack M, Lee H, Shan LB et al. (2010) Differential innate immune signalling via Ca^2+^ sensor protein kinases. Nature 464: 418-422. doi:10.1038/nature08794. PubMed: 20164835. 20164835 PMC2841715

[19] De Vos M, Van Oosten VR, Jander G, Dicke M, Pieterse CMJ (2007) Plants under attack: multiple interactions with insects and microbes. Plant Sig Behav 2: 527-529. doi:10.4161/psb.2.6.4663. PubMed: 19704549. PMC2634359 19704549

[20] Bos JIB, Prince D, Pitino M, Maffei ME, Win J et al. (2010) A functional genomics approach identifies candidate effectors from the aphid species *Myzus* *persicae* (green peach aphid). PLOS Genet 6: e10011216. 10.1371/journal.pgen.1001216 PMC2987835 21124944

[21] Kim JH, Jander G (2007) *Myzus* *persicae* (green peach aphid) feeding on *Arabidopsis* induces the formation of a deterrent indole glucosinolate. Plant J 49: 1008-1019. doi:10.1111/j.1365-313X.2006.03019.x. PubMed: 17257166. 17257166

[22] Pfalz M, Vogel H, Kroymann J (2009) The gene controlling the *INDOLE* *GLUCOSINOLATE* *MODIFIER1* quantitative trait locus alters indole glucosinolate structures and aphid resistance in *Arabidopsis* . Plant Cell 21: 985-999. doi:10.1105/tpc.108.063115. PubMed: 19293369. 19293369 PMC2671713

[23] Clay NK, Adio AM, Denoux C, Jander G, Ausubel FM (2009) Glucosinolate metabolites required for an *Arabidopsis* innate immune response. Science 323: 95-101. doi:10.1126/science.1164627. PubMed: 19095898. 19095898 PMC2630859

[24] Lü B, Sun W, Zhang S, Zhang C, Qian J et al. (2011) HrpN Ea-induced deterrent effect on phloem feeding of the green peach aphid *Myzus* *persicae* requires *AtGSL5* and *AtMYB44* genes in *Arabidopsis* *thaliana* . J Biosci 36: 123-137. doi:10.1007/s12038-011-9016-2. PubMed: 21451254. 21451254

[25] Nishimura MT, Stein M, Hou BH, Vogel JP, Edwards H et al. (2003) Loss of a callose synthase results in salicylic acid-dependent disease resistance. Science 301: 969-972. doi:10.1126/science.1086716. PubMed: 12920300. 12920300

[26] Kettles GJ, Drurey C, Schoonbeek H-J, Maule AJ, Hogenhout SA (2013) Resistance of *Arabidopsis* *thaliana* to the green peach aphid, *Myzus* *persicae*, involves camalexin and is regulated by microRNAs. New Phytol 198: 1178-1190. doi:10.1111/nph.12218. PubMed: 23528052. 23528052 PMC3666093

[27] Glazebrook J, Ausubel FM (1994) Isolation of phytoalexin-deficient mutants of *Arabidopsis* *thaliana* and characterization of their interactions with bacterial pathogens. Proc Natl Acad Sci U S A 91: 8955-8959. doi:10.1073/pnas.91.19.8955. PubMed: 8090752. 8090752 PMC44725

[28] Palukaitis P, García-Arenal F (2003) Cucumoviruses. Adv Virus Res 62: 241-323. PubMed: 14719367. 14719367 10.1016/s0065-3527(03)62005-1

[29] Hayes RJ, Buck KW (1990) Complete replication of a eukaryotic virus RNA *in* *vitro* by a purified RNA-dependent RNA polymerase. Cell 63: 363-368. doi:10.1016/0092-8674(90)90169-F. PubMed: 2208291. 2208291

[30] Suzuki M, Yoshida M, Yoshinuma T, Hibi T (2003) Interaction of replicase components between *Cucumber* *mosaic* *virus* and *Peanut* *stunt* *virus* . J Gen Virol 84: 1931-1939. doi:10.1099/vir.0.19070-0. PubMed: 12810890. 12810890

[31] Lewsey M, Robertson FC, Canto T, Palukaitis P et al. (2007) Selective targeting of miRNA-regulated plant development by a viral counter-silencing protein. Plant J 50: 240-252. doi:10.1111/j.1365-313X.2007.03042.x. PubMed: 17444907. 17444907

[32] Zhang XR, Yuan YR, Pei Y, Lin SS, Tuschl T et al. (2006) Cucumber mosaic virus-encoded 2b suppressor inhibits *Arabidopsis* ARGONAUTE1 cleavage activity to counter plant defense. Genes Dev 20: 3255-3268. doi:10.1101/gad.1495506. PubMed: 17158744. 17158744 PMC1686603

[33] González I, Martínez L, Rakinita DV, Lewsey MG, Atienzo FA et al. (2010) Cucumber mosaic virus 2b protein subcellular targets and interactions: Their significance to RNA silencing suppressor activity. Molecular Plant-Microbe Interact 23: 294-303. doi:10.1094/MPMI-23-3-0294. 20121451

[34] Li Y, Zhang Q, Zhang J, Wu L, Qi Y et al. (2010) Identification of microRNAs involved in pathogen-associated molecular pattern-triggered plant innate immunity. Plant Physiol 152: 2222-2231. doi:10.1104/pp.109.151803. PubMed: 20164210. 20164210 PMC2850012

[35] Robert-Seilaniantz A, MacLean D, Jikumaru Y, Hill L, Yamaguchi S et al. (2011) The microRNA miR393 re-directs secondary metabolite biosynthesis away from camalexin and towards glucosinolates. Plant J 67: 218-231. doi:10.1111/j.1365-313X.2011.04591.x. PubMed: 21457368. 21457368

[36] Lanet E, Delannoy E, Sormani R, Floris M, Brodersen P et al. (2009) Biochemical evidence for translational repression by *Arabidopsis* microRNAs. Plant Cell 21: 1762-1768. doi:10.1105/tpc.108.063412. PubMed: 19531599. 19531599 PMC2714937

[37] Ryabov EV, Fraser G, Mayo MA, Barker H, Taliansky M (2001) Umbravirus gene expression helps *Potato* *leafroll* *virus* to invade mesophyll tissues and to be transmitted mechanically between plants. Virology 286: 363-372. doi:10.1006/viro.2001.0982. PubMed: 11485404. 11485404

[38] Deleris A, Gallego-Bartolome J, Bao J, Kasschau KD, Carrington JC et al. (2006) Hierarchical action and inhibition of plant Dicer-like proteins in antiviral defense. Science 313: 68-71. doi:10.1126/science.1128214. PubMed: 16741077. 16741077

[39] Lewsey MG, Carr JP (2009) Effects of DICER-LIKE proteins 2, 3 and 4 on cucumber mosaic virus and tobacco mosaic virus infections in salicylic acid-treated plants. J Gen Virol 90: 3010-3014. doi:10.1099/vir.0.014555-0. PubMed: 19710258. 19710258

[40] Ziebell H, Carr JP (2009) Effects of DICER-LIKE endoribonucleases 2 and 4 on infection of *Arabidopsis* *thaliana* by cucumber mosaic virus and a mutant virus lacking the 2b counter-defence protein gene. J Gen Virol 90: 2288-2292. doi:10.1099/vir.0.012070-0. PubMed: 19474248. 19474248

[41] González I, Rakitina D, Semashko M, Taliansky M, Palukaitis P et al. (2012) RNA binding is more critical to the suppression of silencing function of *Cucumber* *mosaic* *virus* 2b protein than nuclear localization. RNA 18: 771-782. doi:10.1261/rna.031260.111. PubMed: 22357910. 22357910 PMC3312564

[42] Asaoka R, Shimura H, Arai M, Masuta C (2010) A progeny virus from a *Cucumovirus* pseudorecombinant evolved to gain the ability to accumulate its RNA-silencing suppressor leading to systemic infection in tobacco. Molecular Plant-Microbe Interact 23: 332-339. doi:10.1094/MPMI-23-3-0332. 20121454

[43] Csorba T, Pantaleo V, Burgyán J (2009) RNA silencing: an antiviral mechanism. Adv Virus Res 75: 35-71. PubMed: 20109663. 20109663 10.1016/S0065-3527(09)07502-2

[44] Sisterson MS (2008) Effects of insect-vector preference for healthy or infected plants on pathogen spread: Insights from a model. J Econ Entomol 101: 1-8. Available online at: doi:10.1603/0022-0493(2008)101[1:EOIPFH]2.0.CO;2. PubMed: 18330109. 18330109 10.1603/0022-0493(2008)101[1:eoipfh]2.0.co;2

[45] Weigel D, Mott M (2009) The 1001 Genomes Project for *Arabidopsis* *thaliana* . Genome Biol 10: 107. doi:10.1186/gb-2009-10-10-r107. PubMed: 19519932. 19519932 PMC2718507

[46] Jacquemond M (2012) Cucumber mosaic virus. Adv Virus Res 84: 439-504. PubMed: 22682176. 22682176 10.1016/B978-0-12-394314-9.00013-0

[47] Dawkins R (1982) The extended phenotype: The long reach of the gene. Oxford: Oxford University Press. 307 pp.

[48] Xu P, Chen F, Mannas JP, Feldman T, Sumner LW et al. (2008) Virus infection improves drought tolerance. New Phytol 180: 911-921. doi:10.1111/j.1469-8137.2008.02627.x. PubMed: 18823313. 18823313

[49] Westwood JH, McCann L, Naish M, Dixon H, Murphy AM et al. (2013) A viral RNA silencing suppressor interferes with abscisic acid-mediated signalling and induces drought tolerance in Arabidopsis thaliana. Molecular Plant Pathol 14: 158-170. doi:10.1111/j.1364-3703.2012.00840.x. PMC6638696 23083401

[50] Morel JB, Godon C, Mourrain P, Béclin C, Boutet S et al. (2002) Fertile hypomorphic *ARGONAUTE* (*ago1*) mutants impaired in post-transcriptional gene silencing and virus resistance. Plant Cell 14: 629-639. doi:10.1105/tpc.010358. PubMed: 11910010. 11910010 PMC150585

[51] Harvey JJW, Lewsey MG, Patel K, Westwood J, Heimstädt S et al. (2011) An antiviral defense role of AGO2 in plants. PLOS ONE 6: e14639. doi:10.1371/journal.pone.0014639. PubMed: 21305057. 21305057 PMC3031535

[52] Jacobsen SE, Running MP, Meyerowitz EM (1999) Disruption of an RNA helicase/RNAse III gene in *Arabidopsis* causes unregulated cell division in floral meristems. Development 126: 5231-5243. PubMed: 10556049. 10556049 10.1242/dev.126.23.5231

[53] Schauer SE, Jacobsen SE, Meinke DW, Ray A (2002) DICER-LIKE1: blind men and elephants in *Arabidopsis* development. Trends Plant Sci 7: 487-491. doi:10.1016/S1360-1385(02)02355-5. PubMed: 12417148. 12417148

[54] Devonshire AL, Sawicki RM (1979) Insecticide-resistant *Myzus* *persicae* as an example of evolution by gene duplication. Nature 280: 140-141. doi:10.1038/280140a0.

[55] Banik MT, Zitter TA, Lyons ME (1983) A difference in virus titer of two cucumber mosaic virus isolates as measured by ELISA and aphid transmission. Phytopathol 73: 362.

[56] Roossinck MJ, Palukaitis P (1990) Rapid induction and severity of symptoms in zucchini squash (*Cucurbita* *pepo*) map to RNA1 of cucumber mosaic virus. Molecular Plant-Microbe Interact 3: 188-192. doi:10.1094/MPMI-3-188.

[57] Provvidenti R, Robinson RW, Shail JW (1980) A source of resistance to cucumber mosaic virus in *Lactuca* *saligna* L. for lettuce (L. *sativa* L). Hortsci 15: 528-529

[58] Soards AJ, Murphy AM, Palukaitis P, Carr JP (2002) Virulence and differential local and systemic spread of *Cucumber* *mosaic* *virus* in tobacco are affected by the CMV 2b protein. Mol Plant Microbe Interact 15: 647-653. doi:10.1094/MPMI.2002.15.7.647. PubMed: 12118880. 12118880

[59] Ng JCK, Perry KL (1999) Stability of the aphid transmission phenotype in cucumber mosaic virus. Plant Pathol 48: 388-394. doi:10.1046/j.1365-3059.1999.00348.x.

[60] Zhang L, Handa K, Palukaitis P (1994) Mapping local and systemic symptom determinants of cucumber mosaic cucumovirus in tobacco. J Gen Virol 75: 3185-3191. doi:10.1099/0022-1317-75-11-3185. PubMed: 7964627. 7964627

[61] Rizzo TM, Palukaitis P (1990) Construction of full-length cDNA clones of cucumber mosaic virus RNAs 1, Generation of infectious RNA transcripts. Molec Gen Genet 222: 249-256 10.1007/BF00633825 2274028

[62] Jefferson RA, Kavanagh TA, Bevan MW (1987) GUS fusions: β-glucuronidase as a sensitive and versatile gene fusion marker in higher plants. EMBO J 6: 3901-3907. PubMed: 3327686. 3327686 10.1002/j.1460-2075.1987.tb02730.x PMC553867

[63] Gilliland A, Singh DP, Hayward JM, Moore CA, Murphy AM et al. (2003) Genetic modification of alternative respiration has differential effects on antimycin A-induced versus salicylic acid-induced resistance to *Tobacco* *mosaic* *virus* . Plant Physiol 132: 1518-1528. doi:10.1104/pp.102.017640. PubMed: 12857832. 12857832 PMC167090

[64] Canto T, Uhrig JF, Swanson M, Wright KM, MacFarlane SA (2006) Translocation of *Tomato* *bushy* *stunt* *virus* P19 protein into the nucleus by ALY proteins compromises its silencing suppressor activity. J Virol 80: 9064-9072. doi:10.1128/JVI.00953-06. PubMed: 16940518. 16940518 PMC1563904

[65] Curtis MD, Grossniklaus U (2003) A gateway cloning vector set for high-throughput functional analysis of genes in *planta* . Plant Physiol 133: 462-469. doi:10.1104/pp.103.027979. PubMed: 14555774. 14555774 PMC523872

[66] Clough SJ, Bent AF (1998) Floral dip: A simplified method for *Agrobacterium* mediated transformation of *Arabidopsis* *thaliana* . Plant J 16: 735-743. doi:10.1046/j.1365-313x.1998.00343.x. PubMed: 10069079. 10069079

[67] Carr JP, Gal-On A, Palukaitis P, Zaitlin M (1994) Replicase-mediated resistance to cucumber mosaic virus in transgenic plants involves suppression of both virus replication in the inoculated leaves and long-distance movement. Virology 199: 439-447. doi:10.1006/viro.1994.1142. PubMed: 8122372. 8122372

[68] Canto T, Palukaitis P (1998) Transgenically expressed cucumber mosaic virus RNA 1 simultaneously complements replication of cucumber mosaic virus RNAs 2 and 3 and confers resistance to systemic infection. Virology 250: 325-336. doi:10.1006/viro.1998.9333. PubMed: 9792843. 9792843

[69] Canto T, Prior DAM, Hellwald KH, Oparka KJ, Palukaitis P (1997) Characterization of cucumber mosaic virus. 4. Movement protein and coat protein are both essential for cell-to-cell movement of cucumber mosaic virus. Virology 237: 237-248. doi:10.1006/viro.1997.8804. PubMed: 9356336. 9356336

[70] Leather SR, Dixon AFG (1984) Aphid growth and reproductive rates. Entomologia Experimentalis et Applicata 35: 137-140. doi:10.1111/j.1570-7458.1984.tb03373.x.

[71] Stewart SA, Hodge S, Ismail N, Mansfield JW, Feys BJ et al. (2009) The *RAP1* gene confers effective, race-specific resistance to the pea aphid in *Medicago* *truncatula* independent of the hypersensitive reaction. Mol Plant Microbe Interact 22: 1645-1655. doi:10.1094/MPMI-22-12-1645. PubMed: 19888829. 19888829

[72] Tjallingii WF, Esch Hogen T (1993) Fine-structure of aphid stylet routes in plant-tissues in correlation with EPG signals. Physiol Entomol 18: 317-328. doi:10.1111/j.1365-3032.1993.tb00604.x.

[73] Sarria E, Cid M, Garzo E, Fereres A (2009) Excel workbook for automatic parameter calculation of EPG data. Computers and Electronics in Agriculture 67: 35-42. doi:10.1016/j.compag.2009.02.006.

[74] Hubbell E, Liu WM, Mei R (2002) Robust estimators for expression analysis. Bioinformatics 18: 1585-1592. doi:10.1093/bioinformatics/18.12.1585. PubMed: 12490442. 12490442

[75] Ramakers C, Ruijter JM, Lekanne Deprez RH, Moorman AFM (2003) Assumption-free analysis of quantitative real-time polymerase chain reaction (PCR) data. Neurosci Lett 339: 62-66. doi:10.1016/S0304-3940(02)01423-4. PubMed: 12618301. 12618301

[76] Ruijter JM, Ramakers C, Hoogaars WMH, Karlen Y, Bakker O et al. (2009) Amplification efficiency: linking baseline and bias in the analysis of quantitative PCR data. Nucleic Acids Res 37: e45. doi:10.1093/nar/gkp045. PubMed: 19237396. 19237396 PMC2665230

[77] Yuan JS, Wang D, Stewart CNJ (2008) Statistical methods for efficiency adjusted real-time PCR quantification. Biotechnol J 3: 112-123 10.1002/biot.200700169 18074404

[78] Rossiter JT, James DC, Atkins N (1990) Biosynthesis of 2-hydroxy-3-butenylglucosinolate and 3-butenylglucosinolate in *Brassica* *napus* . Phytochemistry 29: 2509-2512. doi:10.1016/0031-9422(90)85177-H.

[79] Brown PD, Tokuhisa JG, Reichelt M, Gershenzon J (2003) Variation of glucosinolate accumulation among different organs and developmental stages of *Arabidopsis* *thaliana* . Phytochemistry 62: 471-481. doi:10.1016/S0031-9422(02)00549-6. PubMed: 12620360. 12620360

[80] Surplus SL, Jordan BR, Murphy AM, Carr JP, Mackerness SA-H (1998) Ultraviolet-B-induced responses in *Arabidopsis* *thaliana*: role of salicylic acid and reactive oxygen species in the regulation of transcripts encoding photosynthetic and acidic pathogenesis-related proteins. Plant, Cell Environ 21: 685-694. doi:10.1046/j.1365-3040.1998.00325.x.

[81] Anderson TW, Darling DA (1952) Asymptotic theory of certain "goodness-of-fit" criteria based on stochastic processes. Annals Math Stat 23: 193-212

[82] Johnson NJ (1978) Modified t tests and confidence intervals for asymmetrical populations. J American Stat Assoc 73: 536-544. doi:10.2307/2286597.

[83] Chou YM, Polansky A, Mason RL (1998) Transforming non-normal data to normality in statistical process control. J Quality Technol 30: 133-141.

[84] Wan J, Zhang XC, Neece D, Ramonell KM, Clough S et al. (2008) A LysM receptor-like kinase plays a critical role in chitin signaling and fungal resistance in *Arabidopsis* . Plant Cell 20: 471-481. doi:10.1105/tpc.107.056754. PubMed: 18263776. 18263776 PMC2276435

[85] Bartsch M, Gobbato E, Bednarek P, Debey S, Schultze JL et al. (2006) Salicylic acid-independent ENHANCED DISEASE SUSCEPTIBILITY1 signaling in *Arabidopsis* immunity and cell death is regulated by the monooxygenase FMO1 and the nudix hydrolase NUDT7. Plant Cell 18: 1038-1051. doi:10.1105/tpc.105.039982. PubMed: 16531493. 16531493 PMC1425861

