# Supplementary figures and images for: A Trio of Viral Proteins Tunes Aphid-Plant Interactions in Arabidopsis thaliana
Source: PLoS One. 2013 Dec 11;8(12):e83066. doi: 10.1371/journal.pone.0083066 (PMC3859657; doi:10.1371/journal.pone.0083066)

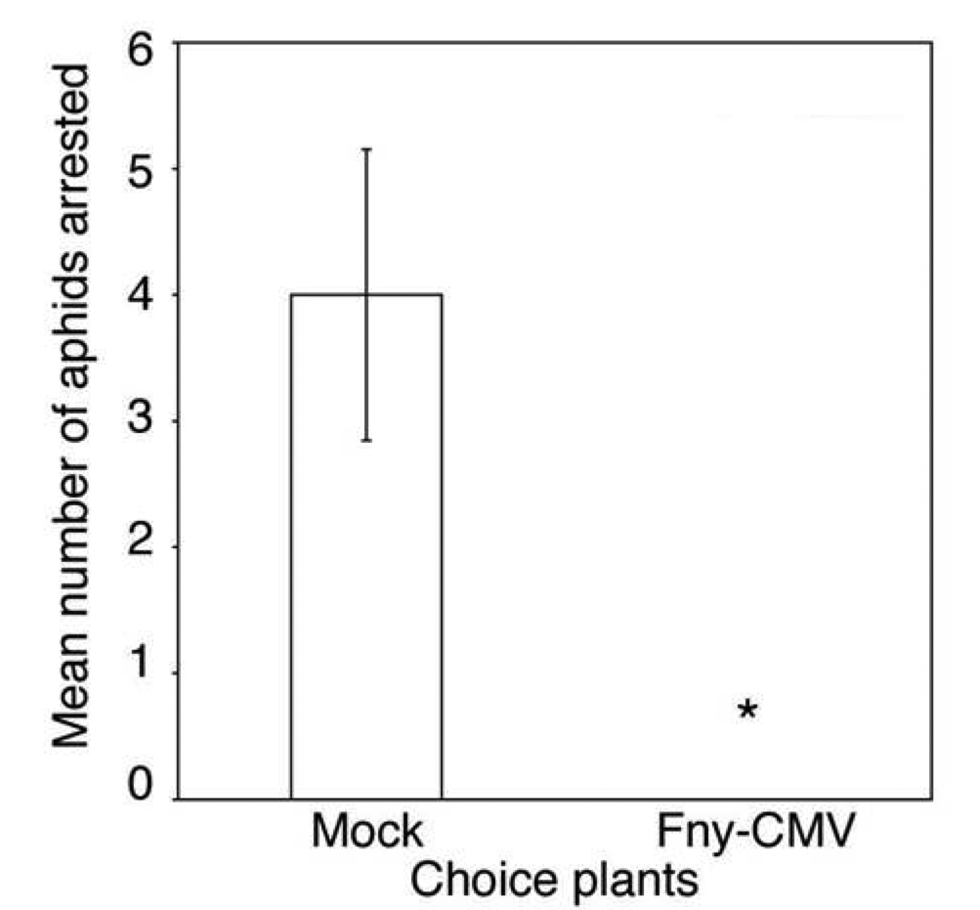

Supplement: Figure S1 — Aphid migration behavior is enhanced by CMV infection of wild-type Arabidopsis plants. Ten aphids were released onto rosettes of mock-inoculated or Fny-CMV-infected release plants and then allowed to remain or emigrate to a plant of the opposite treatment group located 10 cm away in the same pot. Aphids migrated away more often from Fny-CMV-infected than from mock-inoculated plants. Fny-CMV infected choice plants arrested fewer aphids at 24 hours relative to mock-inoculated plants. Based on the methods of Mauck et al. [8], three independent tests were performed for each type of release plant. See Figure 1A for the accompanying aphid migration data. Error bars represent standard error of the mean. Asterisks indicate significant differences (Student’s t-test): *, P<0.05; **, P<0.01; ***, P<0.001. (TIF) [file pone.0083066.s001.tif]

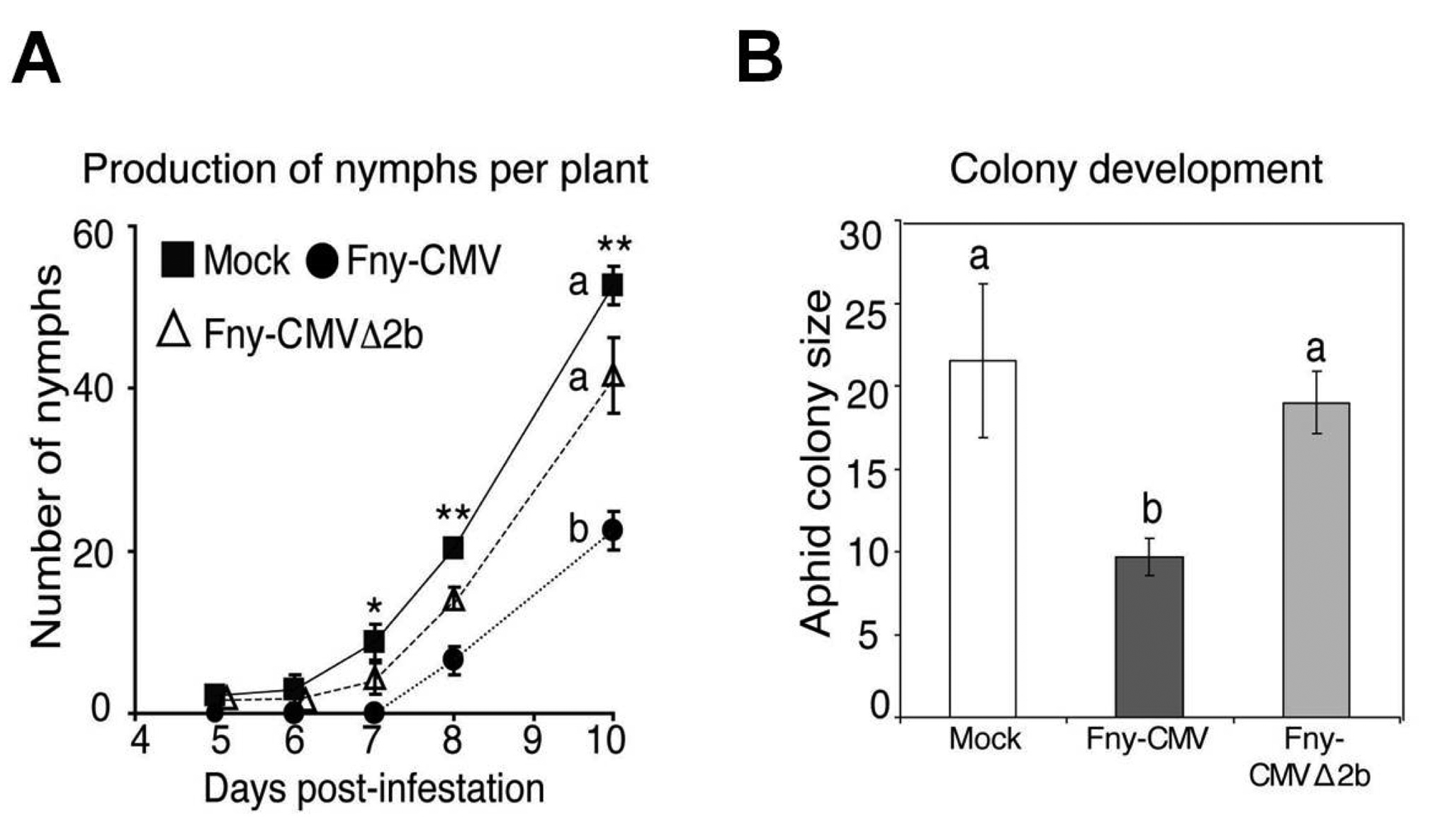

Supplement: Figure S2 — Aphid behavior and performance on virus-infected wild-type plants. (A) Production of aphid nymphs from an initial infestation of two one-day-old nymphs (per plant) was monitored over a 10 day period, n=6. Statistically significant (ANOVA with post-hoc Tukey’s tests) differences are indicated: *, P<0.05, and **, P<0.001. (B) Aphid colony size produced from initial infestations of single one-day-old nymph at 14 days post-infestation, n≥10. Different letters are assigned to significantly different groups (ANOVA with post-hoc Tukey’s tests, P<0.05). Error bars represent standard error of the mean. (TIF) [file pone.0083066.s002.tif]

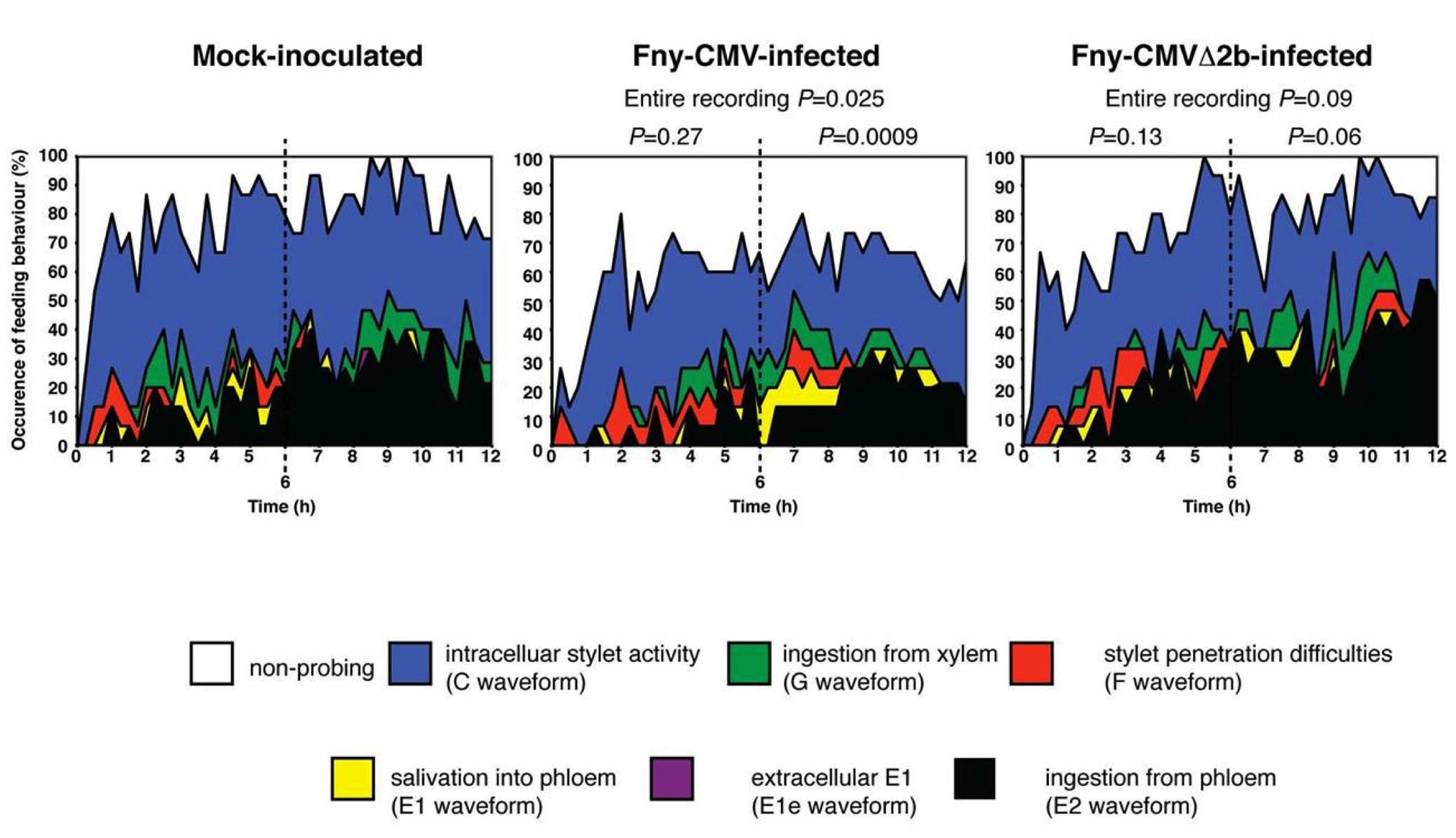

Supplement: Figure S3 — Percentage occurrence of waveforms produced in electrical penetration graph (EPG) analysis of feeding aphids over 12-hour recordings. Ingestion from the phloem (E2 waveform; black) was significantly decreased for aphids feeding on Fny-CMV-infected plants. Statistical significance was tested by Student’s t-test compared to percentage occurrence of phloem ingestion on mock-inoculated plants for the first and second halves of the recording and for the whole recording. (TIF) [file pone.0083066.s003.tif]

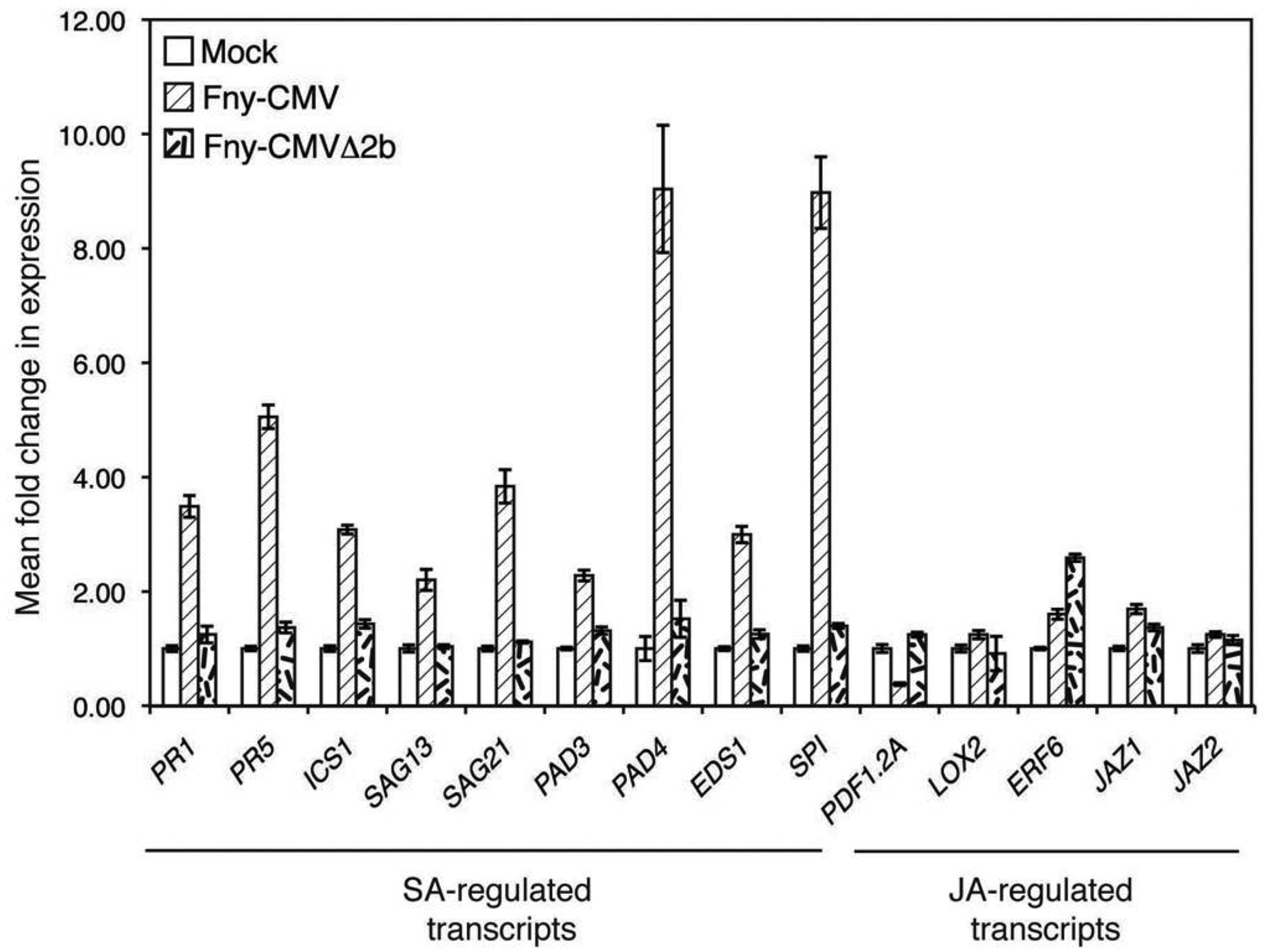

Supplement: Figure S4 — Confirmation of the responses of selected salicylic acid- and jasmonic acid-regulated transcripts to Fny-CMV and Fny-CMVΔ2b infection by RT-Q-PCR at 14 days post-inoculation. Mean fold change in expression was calculated relative to the expression of each gene in mock-inoculated plants. Error bars represent standard error of the mean. (TIF) [file pone.0083066.s004.tif]

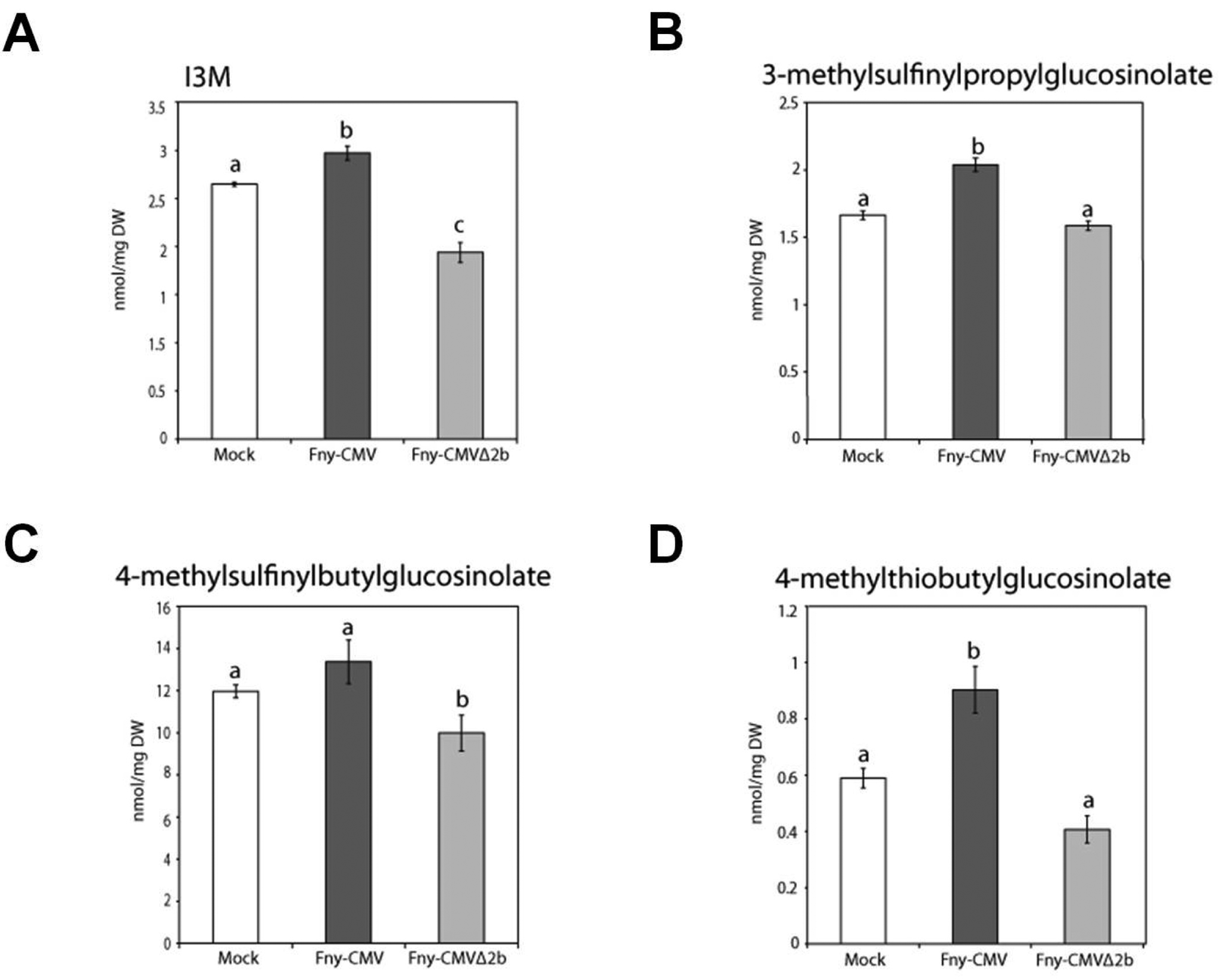

Supplement: Figure S5 — Glucosinolate accumulation in virus-infected wild-type plants. (A-D) Glucosinolate accumulation in Fny-CMV- and Fny-CMVΔ2b-infected wild type plants was analyzed using reverse phase high performance liquid chromatography. Error bars represent standard error of the mean. Abbreviation: I3M, indol-3-yl-methylglucosinolate. Different letters are assigned to significantly different results (ANOVA with post-hoc Tukey’s tests P<0.05). (TIF) [file pone.0083066.s005.tif]

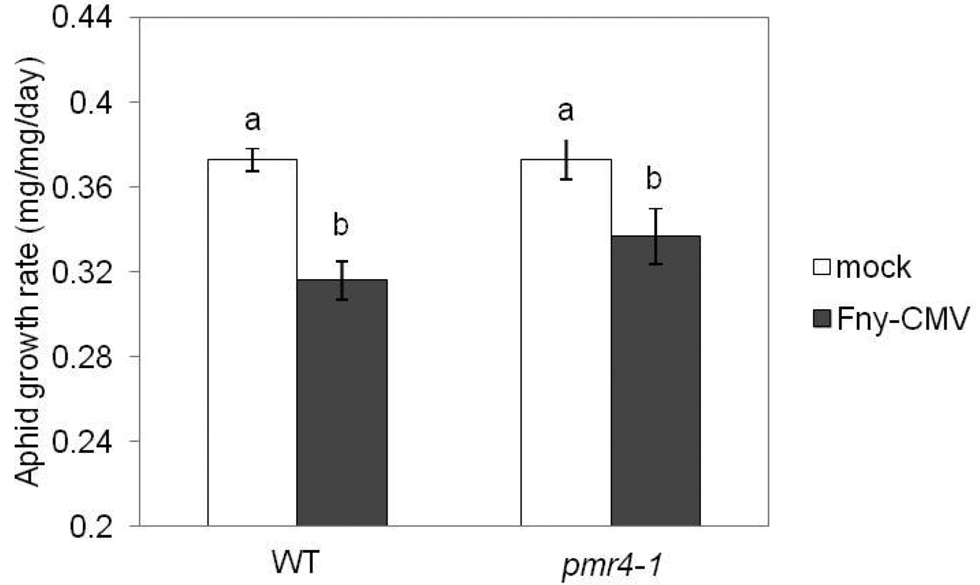

Supplement: Figure S6 — Aphid performance on virus-infected wild-type and pmr4-1 mutant plants. Mean relative growth rate of individual aphids feeding on pmr4-1 mutant (which cannot accumulate callose) and wild type (WT) plants, n≥24. Error bars represent standard error of the mean. Different letters are assigned to significantly different groups (ANOVA with post-hoc Tukey’s tests, P<0.05). (TIF) [file pone.0083066.s006.tif]

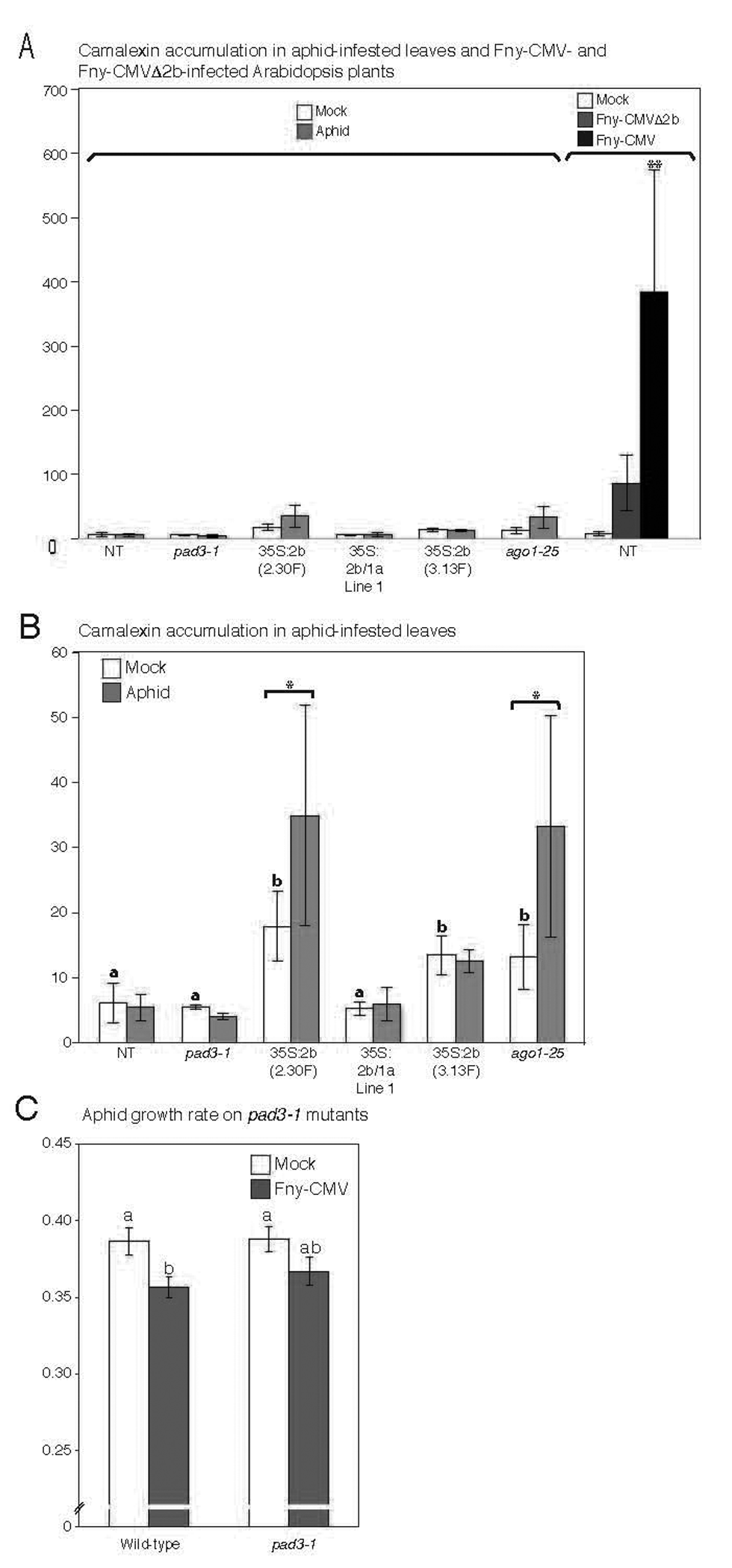

Supplement: Figure S7 — Camalexin does not play a major role in Fny-CMV-induced resistance to Mysus persicae in Arabidopsis. (A) Differences in camalexin accumulation in Arabidopsis leaves 48 hours post-infestation with 30 aphid nymphs were small compared to leaves systemically infected with Fny-CMV or Fny-CMVΔ2b, n=3. Statistically significant (ANOVA with post-hoc Tukey’s tests) differences are indicated: **, P<0.001. See panel (B) for statistics on the aphid-infested plants. (B) Detailed examination of camalexin accumulation in aphid-infested leaves [also shown in panel (A)] revealed that in plants with disrupted miRNA utilization (35S:2b-expressing lines 2.30F and 3.13F and ago1-25), basal levels of camalexin were significantly higher (ANOVA with post-hoc Tukey’s tests, P<0.01) than in non-transformed (NT) plants and pad3-1 mutant plants. Interestingly, the double transgenic plants expressing the 2b and 1a proteins had levels of camalexin similar to NT plants. Aphid infestation resulted in significantly increased levels of camalexin relative to the mock-treatment in the 35S:2b-expressing line 2.30F and in the mutant ago1-25 (ANOVA with post-hoc Tukey’s tests, P<0.05). (C) The mean relative growth rate of individual aphids feeding on Fny-CMV-infected wild-type and pad3-1 mutants (impaired in camalexin biosynthesis) was lower compared to aphids feeding on mock-inoculated plants, n≥24. The decrease in aphid growth rate on Fny-CMV-infected pad3-1 mutants was slightly less severe than in virus-infected wild-type plants (ANOVA with post-hoc Tukey’s tests, P<0.05) indicating that camalexin can make only a minor contribution at best to aphid resistance induced by Fny-CMV. Error bars represent standard error of the mean. Different letters are assigned to significantly different groups. (TIF) [file pone.0083066.s007.tif]

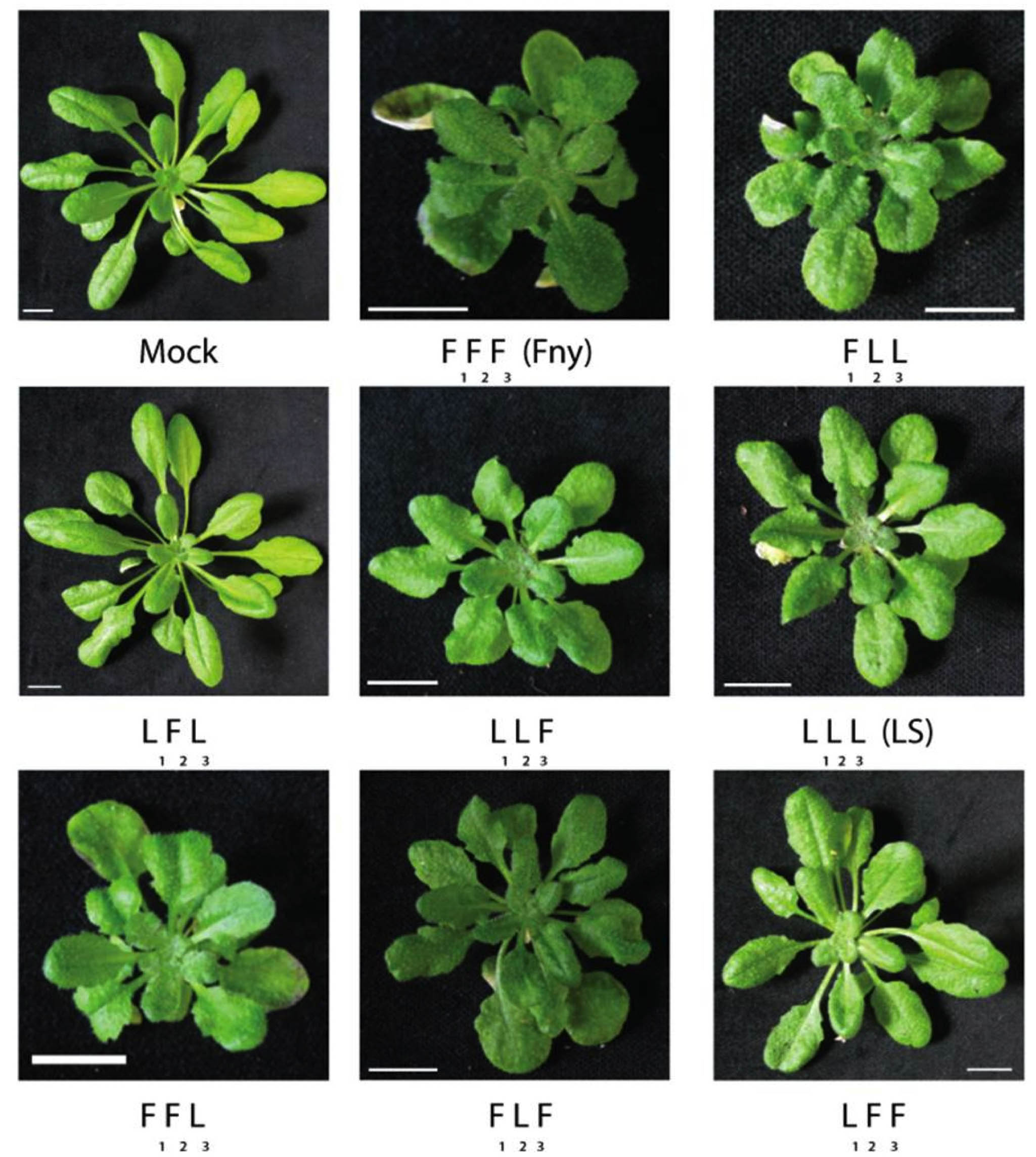

Supplement: Figure S8 — Symptoms induced by CMV reassortants in wild-type Arabidopsis plants. Viral reassortants generated using combinations of CMV genomic RNAs 1, 2 or 3 (subscript) from either Fny-CMV (F) or LS-CMV (L) were used to inoculate wild-type Arabidopsis plants (Col-0 ecotype). Mock indicates a mock-inoculated plant. Plants were photographed at 14 days post-inoculation. Scale bars represent 1 cm. (TIF) [file pone.0083066.s008.tif]

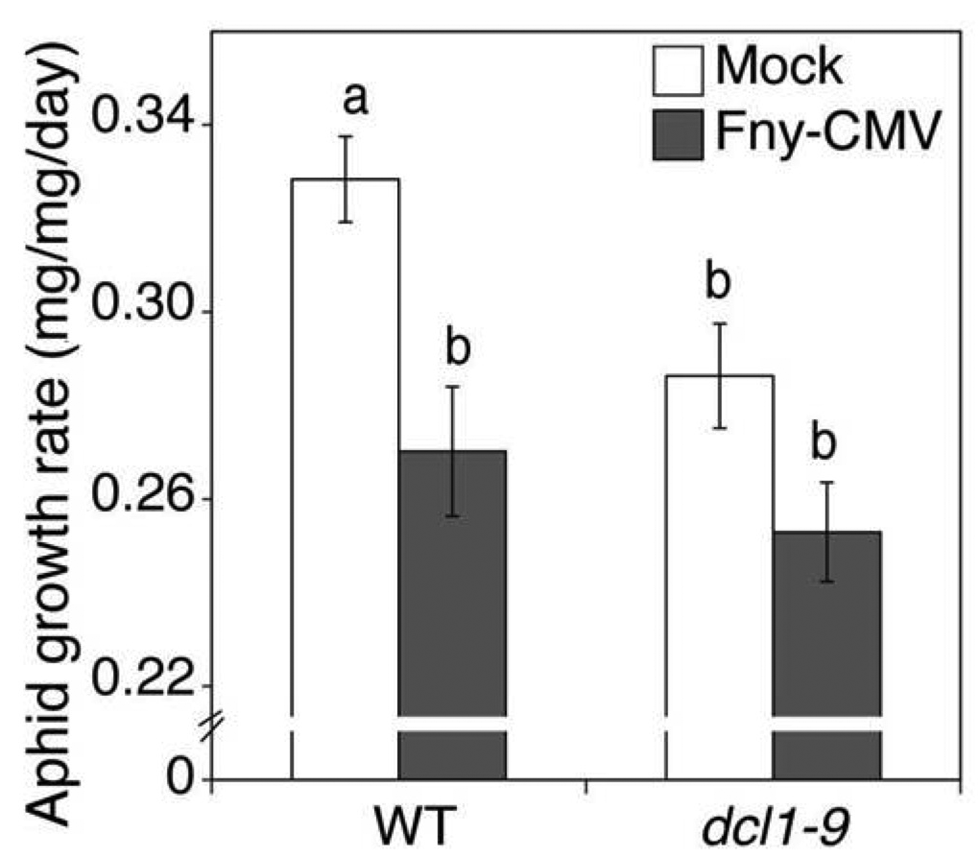

Supplement: Figure S9 — Aphid performance on virus-infected wild-type plants and dcl1-9 mutant plants. Mean relative growth rate of individual aphids feeding on dcl1-9 mutant and wild type (WT) Arabidopsis plants, n≥24. Error bars represent standard error of the mean. Different letters are assigned to significantly different groups (ANOVA with post-hoc Tukey’s tests, P<0.05). (TIF) [file pone.0083066.s009.tif]

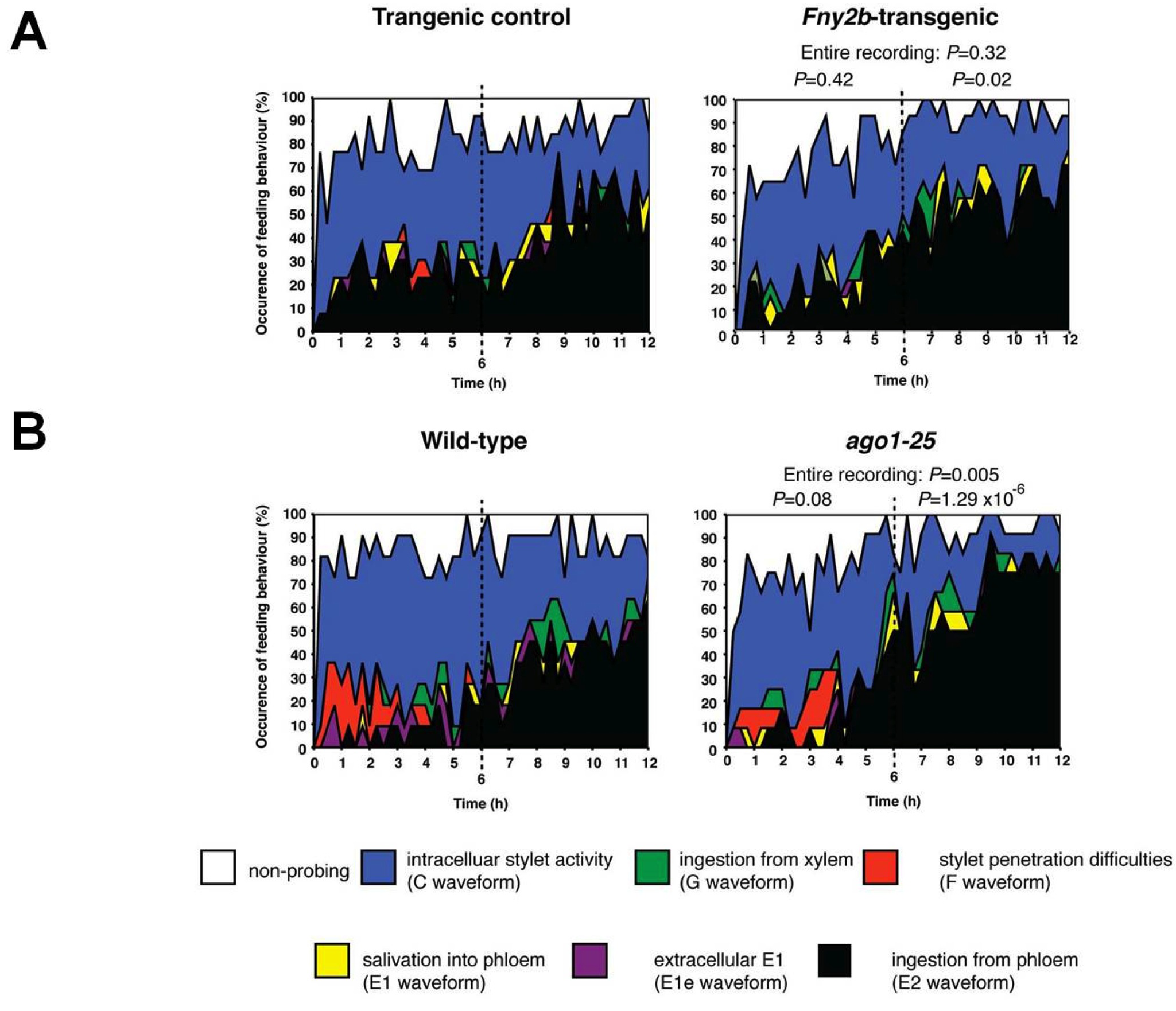

Supplement: Figure S10 — Percentage occurrence of waveforms produced in electrical penetration graph (EPG) analysis of feeding aphids over 12-hour recordings. (A) Ingestion from the phloem (E2 waveform, colored black) was significantly increased for aphids feeding on Fny2b-transgenic plants in the second half of recordings. (B) Ingestion from the phloem (E2 waveform, colored black) was significantly increased for aphids feeding on ago1-25 transgenic plants across the whole recording. Statistical significance was tested by Student’s t-test compared to percentage occurrence of phloem ingestion on mock-inoculated plants for the first and second halves of the recording and for the whole recording. (TIF) [file pone.0083066.s010.tif]

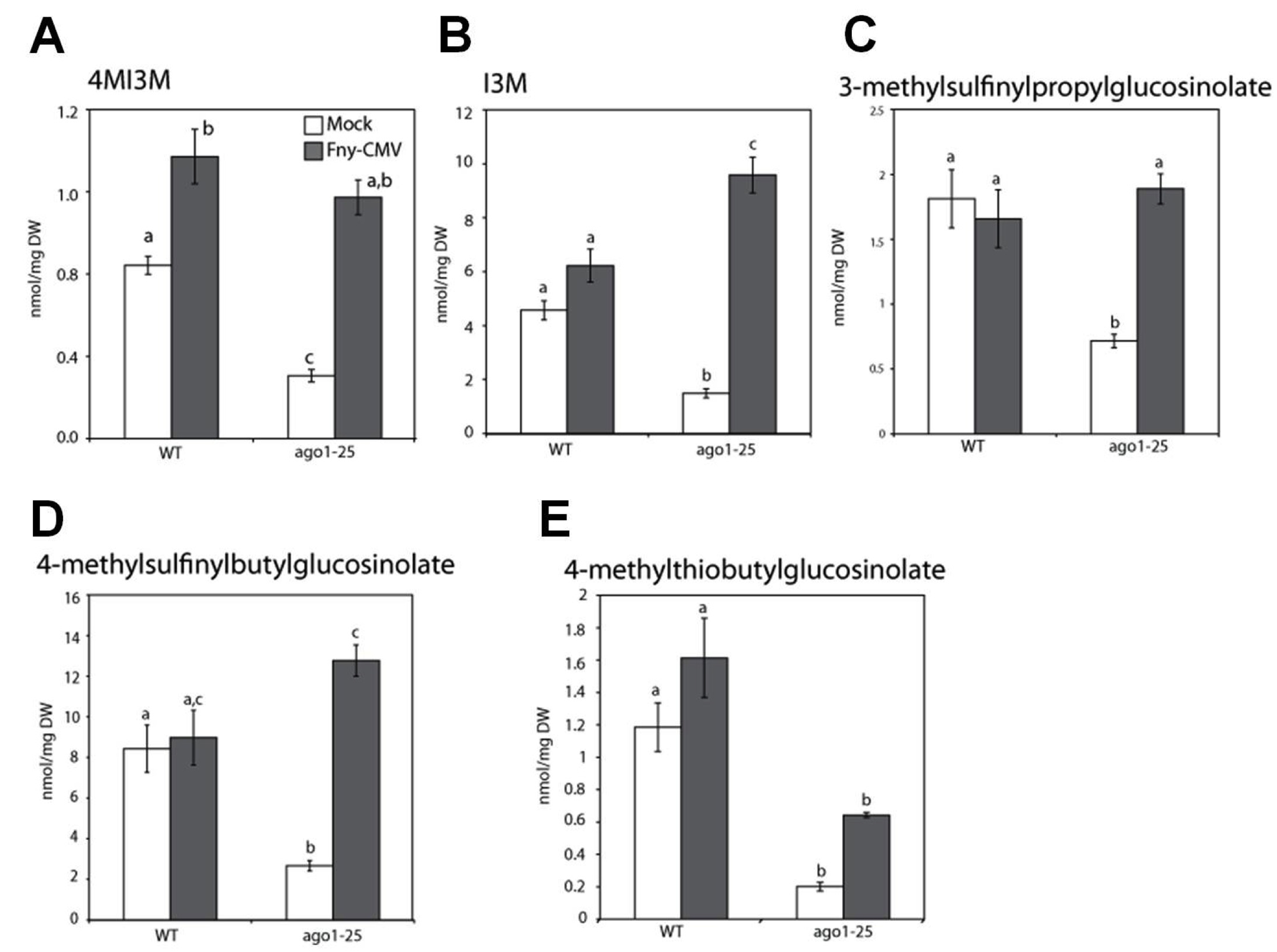

Supplement: Figure S11 — Glucosinolate accumulation in wild-type plants and ago1-25 mutants. Glucosinolate accumulation of Fny-CMV-infected wild-type plants and ago1-25 mutants was analyzed using high performance liquid chromatography, n=3. Error bars represent standard error of the mean. Abbreviations: 4MI3M, 4-methoxy-indol3yl-methylglucosinolate, and I3M, indol-3-yl-methylglucosinolate. Different letters are assigned to significantly different results (ANOVA with post-hoc Tukey’s tests P<0.05). (TIF) [file pone.0083066.s011.tif]

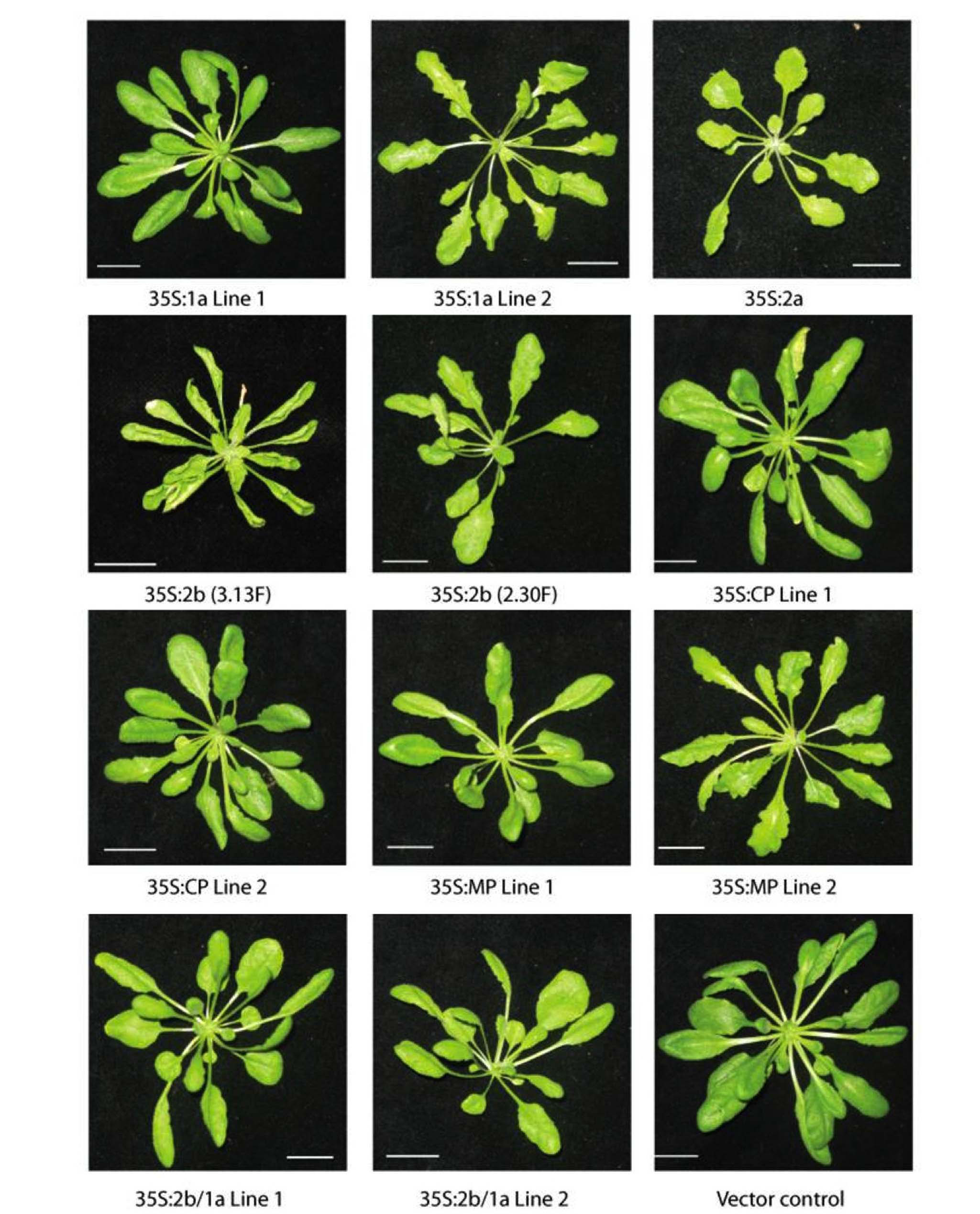

Supplement: Figure S12 — Phenotypes of transgenic Arabidopsis plants constitutively expressing various Fny-CMV proteins. Appearance of plants (from independent transformed lines) expressing transgenes encoding the CMV proteins 1a, 2a, 2b, movement protein (MP), and coat protein (CP) under the control of the constitutive cauliflower mosaic virus 35S promoter. Vector control is a plant from a line transformed with ‘empty’ pBI121.1. Plants were five weeks old when photographed. The 2b-transgenic lines 2.30F and 3.13F were constructed by Lewsey and colleagues [31]. Scale bar represents 1 cm. (TIF) [file pone.0083066.s012.tif]

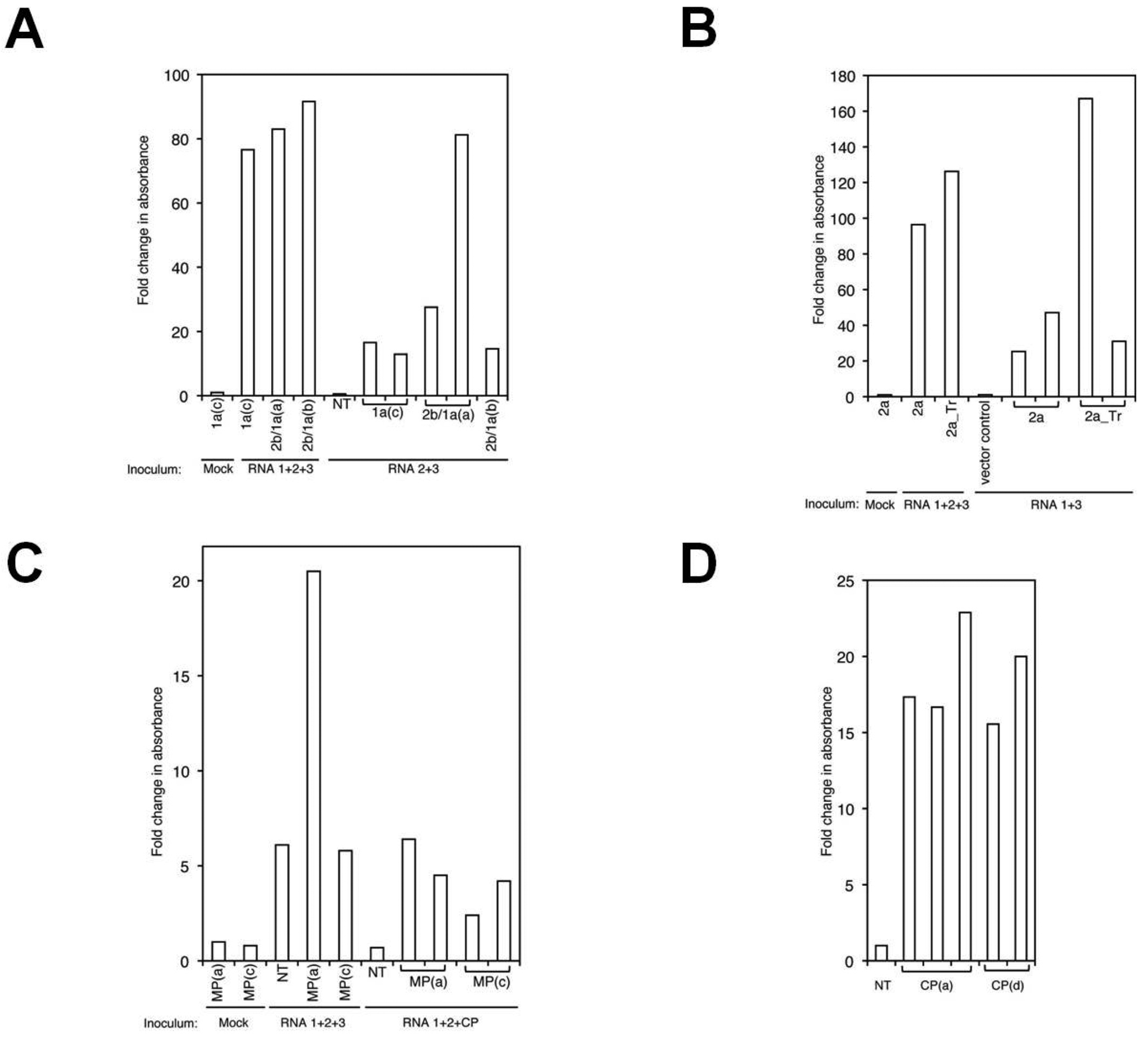

Supplement: Figure S13 — CMV proteins transgenically expressed in Arabidopsis are biologically active. (A) The transgenic plants expressing the 1a [1a (c)] or 2b and 1a [2b/1a (a) and 2b/1a (b)] ORFs of Fny-CMV were inoculated with a mixture of synthetic RNAs 2 and 3 (RNA 2+3) generated by in vitro transcription of the plasmids pFny209 and pFny309, respectively. Three weeks post-inoculation, CMV coat protein (CP) was detected by DAS-ELISA in the non-inoculated leaves indicating that virus replication had occurred (due to complementation of efficient replication and spread by the transgenically-expressed viral protein). (B) Transgenic plants expressing the 2a (2a) and truncated 2a (2a_Tr) ORFs were inoculated with synthetic RNAs 1 and 3 (RNA 1+3) generated by in vitro transcription of the clones pFny109 and pFny309, respectively. Three weeks post inoculation CMV CP was detected by DAS-ELISA in the non-inoculated leaves indicating that virus replication had occurred and therefore that the 2a-derived transgenes were expressing enzymatically active 2a (RNA-dependent RNA polymerase) protein and complementing efficient viral replication and spread. (C) Transgenic plants expressing the MP gene [independent lines MP (a) and MP (c)] were inoculated with RNA 1 and 2 and a modified RNA 3 containing the GFP gene in place of the MP ORF (RNA 1+2+CP), where CP represents the synthetic RNA transcribed from pF:GFP/CP as described previously (58,69). Three weeks post-inoculation, CMV CP was detected by DAS-ELISA in the non-inoculated leaves indicating that the transgenically expressed MP had complemented cell-to-cell movement and facilitated systemic movement of RNAs 1, 2 and the modified RNA 3. (D) Expression of CP was directly detected by DAS-ELISA in leaf tissue from transgenic plants expressing the CP ORF. Additional transgenic plants in (A-C) have been inoculated with a mixture of RNAs 1, 2, and 3 (RNA1+2+3), which constitutes the whole Fny-CMV genome, as a positive control. Non-transgenic (NT) plants a [file pone.0083066.s013.tif]

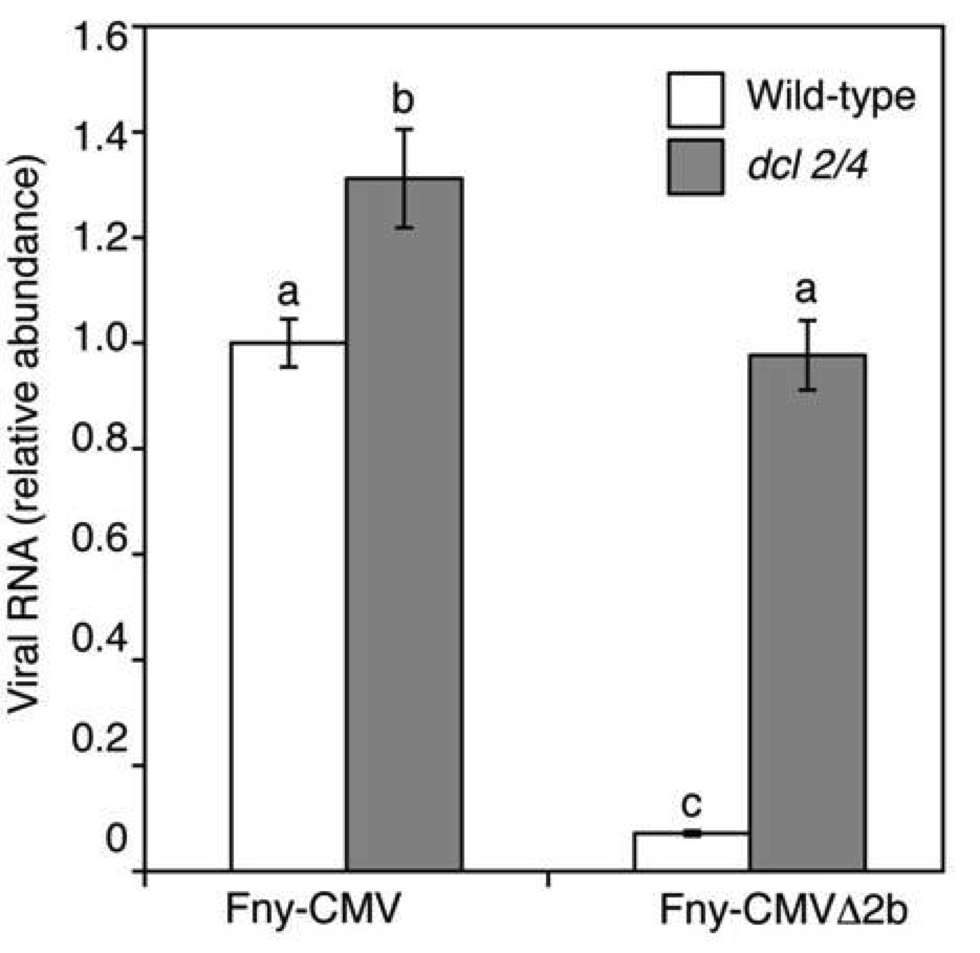

Supplement: Figure S14 — Relative accumulation of Fny-CMV and Fny-CMVΔ2b in wild-type and dcl2/4 double mutant plants. Viral RNA accumulation was measured (by RT-Q-PCR) relative to the accumulation of Fny-CMV RNA in wild-type plants at 14 days post-inoculation, n=3. Error bars represent standard error of the mean. the experiments was repeated three times with similar results. Different letters are assigned to statistically different groups (ANOVA with post-hoc Tukey’s tests, P<0.05). (TIF) [file pone.0083066.s014.tif]

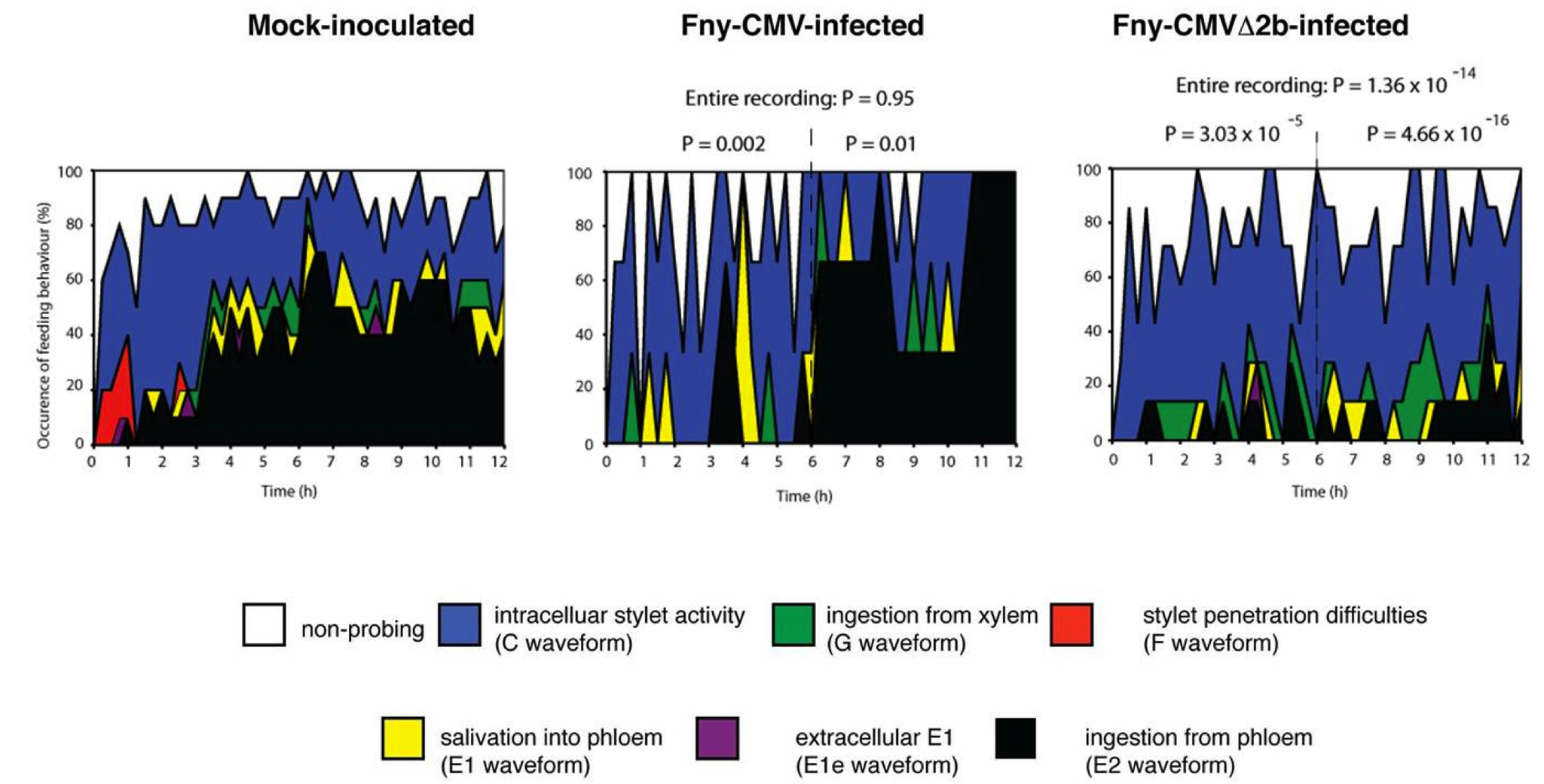

Supplement: Figure S15 — Aphid feeding behavior on dcl2/4 mutant plants: Percentage occurrence of waveforms produced in electrical penetration graph (EPG) analysis of feeding aphids over 12-hour recordings on virus infected dcl2/4 mutants. Ingestion from the phloem (E2 waveform, colored black) was significantly increased in the second half of the recording for aphids feeding on Fny-CMV infected dcl2/4 mutant plants. Ingestion from the phloem was significantly decreased for aphids feeding on Fny-CMVΔ2b-infected dcl2/4 mutant plants. Statistical significance was tested by Student’s t-test compared to percentage occurrence of phloem ingestion on mock-inoculated plants for the first and second halves of the recording and for the whole recording. (TIF) [file pone.0083066.s015.tif]

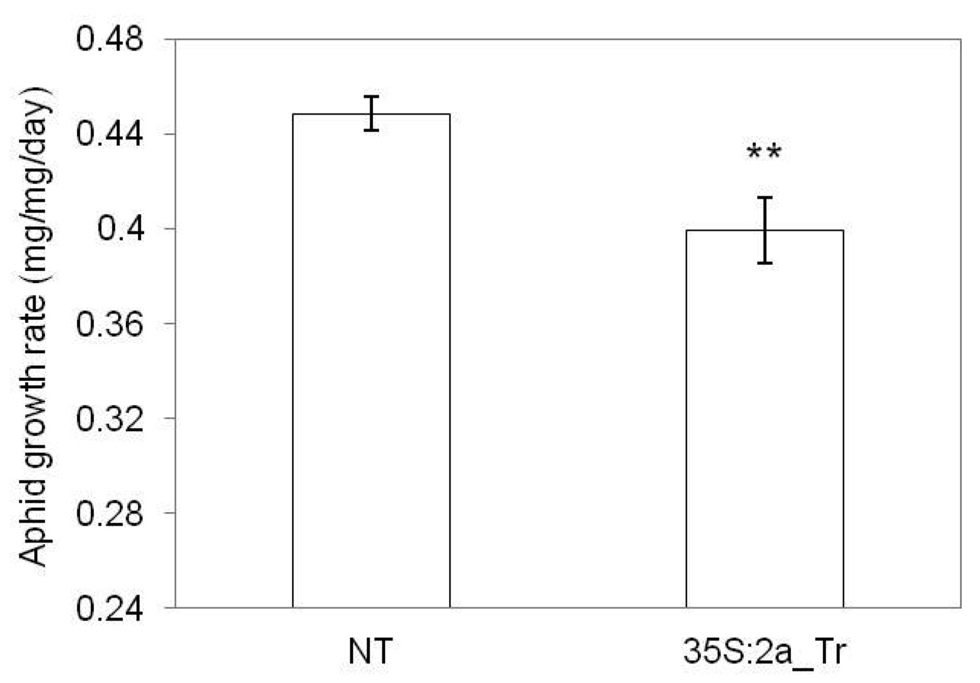

Supplement: Figure S16 — A truncated form of the Fny2a protein had the capacity to induce anti-aphid resistance. Growth rate of individual aphids feeding on transgenic plants expressing a truncated form of the Fny2a protein (35S:2a_Tr) and non-transgenic (NT) plants, n≥24. Error bars represent standard error of the mean. Asterisks indicate significant differences (Student’s t-test): *, P<0.05; **, P<0.01; ***, P<0.001. (TIF) [file pone.0083066.s016.tif]

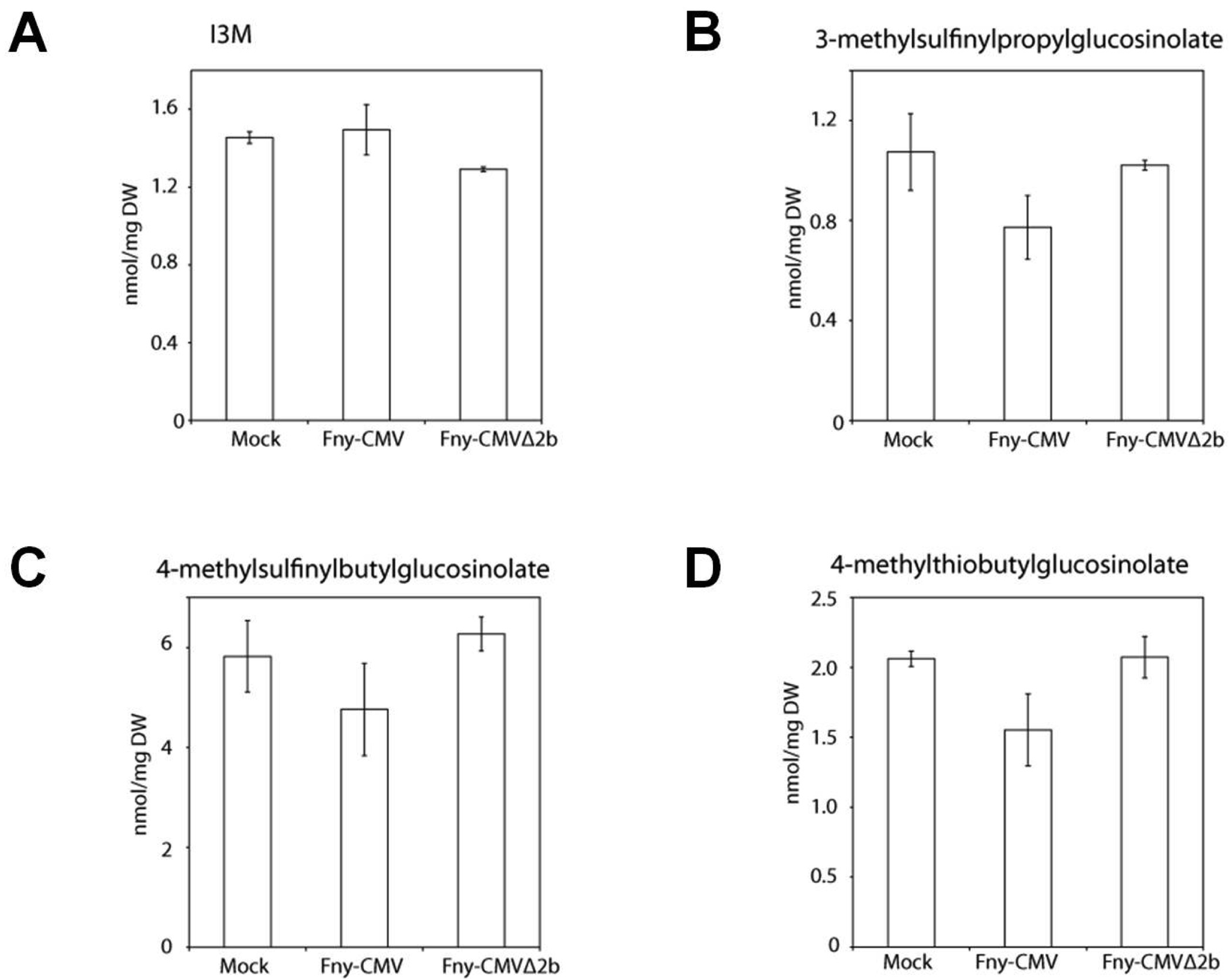

Supplement: Figure S17 — High performance liquid chromatography analysis of glucosinolate accumulation in Fny-CMV-infected (Fny-CMV) and Fny-CMVΔ2b-infected (Fny-CMVΔ2b) dcl2/4 double mutant plants. Statistical analysis did not reveal any significant differences compared to mock-inoculated plants (Mock) (ANOVA with post-hoc Tukey’s tests, P>0.05), except for the increased accumulation of 4-methoxy-indol-3-yl-methylglucosinolate in Fny-CMVΔ2b-infected dcl2/4 double mutants (see Figure 9D of the main text). Error bars represent standard error of the mean. Data presented represents the mean accumulation of each glucosinolate extracted from tissue from at least three plants per treatment grouped and repeated independently three times. (TIF) [file pone.0083066.s017.tif]
