# Supplementary material for: A Trio of Viral Proteins Tunes Aphid-Plant Interactions in Arabidopsis thaliana
Source: PLoS One. 2013 Dec 11;8(12):e83066. doi: 10.1371/journal.pone.0083066 (PMC3859657; doi:10.1371/journal.pone.0083066)
Supplement: Table S1 — Primers used in generation of transgenic plants expressing proteins derived from Fny-CMV. (DOC) [file pone.0083066.s021.doc]

Supplementary Table 1. Primers used in generation of transgenic plants expressing proteins derived from Fny-CMV

| Target ORF | Primers | | Destination Vector | Selection *in planta* |
| --- | --- | --- | --- | --- |
|  | Forward Primer | Reverse Primer |  |  |
| 1a | tatggatccatggcgacgtcctcgttc | gagagctcctagtgatggtgatgatcagcacgagcaacacattcgg | pBI121 | Kan |
|  | tatggtaccatggcgacgtcctcgttc | gagctagcgtagtgatggtgatgatgagcacgagcaacacattcgg | pDJSn | Hyg |
| 2a^§^ | aaaaagcaggctacatggctttccctgcccccgcattctcactag | agaaagctgggtctcagtgatggtgatgatggactcgggtaactcc | pLX221 | Kan |
| 2atrunc^§^ |  | agaaagctgggtctcagtgatggtgatgatgattctttcgctgtttg | pLX221, PMDC45 | Kan  Hyg |
| MP | tatggatccatggctttccaaggtaccag | gagagctctcagtgatggtgatgatggataagaccgttaaccacctg | pBI121 | Kan |
| CP | tatggatccatggacaaatctgaatcaac | gagagctctcagtgatggtgatgatggatcgggagcactccagatg | pBI121 | Kan |

^§^Products from these reactions were subsequently used as templates in PCR using primers to generate a full ‘att’ site either side of the ORF (F- ggggacaagtttgtacaaaaaagcaggct, R-ggggaccactttgtacaagaaagctgggt) allowing recombination into the pDONR207 vector followed by recombination into the compatible gateway binary vectors pLXR221 and pMDC.
