# Supplementary material for: A Trio of Viral Proteins Tunes Aphid-Plant Interactions in Arabidopsis thaliana
Source: PLoS One. 2013 Dec 11;8(12):e83066. doi: 10.1371/journal.pone.0083066 (PMC3859657; doi:10.1371/journal.pone.0083066)
Supplement: Table S2 — Primers used in RT-Q-PCR analyses. ELONGATION FACTOR 1α (EF1α) and GAPDH were used as stable reference transcripts to control for loading. (DOC) [file pone.0083066.s022.doc]

**Table S2. Primers used in RT-Q-PCR analyses.** *ELONGATION FACTOR 1α* (EF1*α*) and *GAPDH* were used as stable reference transcripts to control for loading.

| **Transcript** | **Locus ID** |  | **Sequence (5' to 3')** |
| --- | --- | --- | --- |
| *EF1α* | AT5G60390 | F | CGGTGCCAGTGGGACGTGTT |
|  |  | R | TGGCGGCACCCTTAGCTGGA |
| *GAPDH* | AT1G13440 | F | aggctgggattgcattgagcga |
|  |  | R | acacacaaactctcgccggtgt |
| *PAD4* | AT3G52430 | F | TGGGCTTGCCAGTCACCGGA |
|  |  | R | AAGACGCGGAATGACGGCGG |
| *SAG13* | AT2G29350 | F | AGCCTCTGGCTCAGGGAGCA |
|  |  | R | TGTCGCTCGCCCATTCGCAA |
| *SAG21* | AT4G02380 | F | ACGTGGTTATGCGGCCACGG |
|  |  | R | TGCTCGTAGCTCAGCCGCGT |
| *PR1* | AT2G14610 | F | CGAAAGCTCAAGATAGCCCA |
|  |  | R | AAGGCCCACCAGAGTGTATG |
| *ICS1* | AT1G74710 | F | TATCTCCGGCAGCCGCCACT |
|  |  | R | CGGGACGACCAACGTCACTGC |
| *PDF1.2a* | AT5G44420 | F | TGCTTTCGACGCACCGGCAA |
|  |  | R | TGCATTACTGTTTCCGCAAACCCC |
| *ERF6* | AT4G17490 | F | TCGCACCACCGAACCGAACC |
|  |  | R | GCGAATTTCCCCCACGGCCT |
| *JAZ1* | AT1G19180 | F | CCGGGCAAGTGATTGTATTC |
|  |  | R | TGCGATAGTAGCGATGTTGC |
| *JAZ2* | AT1G74950 | F | TCGGTTCCTTGAGAAGAGGA |
|  |  | R | GACAGGCTCGAAGTTTACCG |
| *JAZ7* | AT2G34600 | F | GCTCGTTGGACGAATCAAGCAGC |
|  |  | R | TGTTGGAGGATCCGAACCGTCTG |
| *LOX2* | AT3G45140 | F | ATACCCATCGCAGAAACAGG |
|  |  | R | TGGAGCTTGCCTTTGAATCT |
| *VSP2* | AT5G24770 | F | CGGGGGCGTACTGGTTGTG |
|  |  | R | CCTCAAGTTCGAACCATTAGGCTTCA |
| *PAD3* | AT3G26830 | F | GACCAGAGGCGGCCGGAATG |
|  |  | R | CGACCACCGAGAGCTTCCGC |
| *SPI* | AT1G72060 | F | GGTGATGTCTTTCATCATAGCAGGAGC |
|  |  | R | TGTCAGCGCCGGAGGAAGGA |
| *EDS1* | AT3G48090 | F | TGACTGGAAGCGCAGAGGCG |
|  |  | R | TGGCGTCCGAGTTGTTCACTG |
| *PR5* | AT1G75040 | F | CGCCGGTCAAGGACCCAAGC |
|  |  | R | AGACCGCCACAGTCTCCGGT |
| *MPK3* | At3G45650 | F | AGTTGCTTGGCACACCGACAGA |
|  |  | R | AAGGGCTGACGTGGGAAGTTGG |
| *FRK1* | AT2G19190 | F | TGACGTTGGCTCGGCTTGGA |
|  |  | R | TGCTCGAGGAACCATCTCGGTG |
| *CYP81F2* | AT5G57220 | F | AGTGAACGCTTGGGCCATCCAT |
|  |  | R | GGCCTAAAGTCGCACCAGGACA |
| *AGO2* | AT1G31280 | F | ATCGTGGTCAAGGTCGTGGTGAAC |
|  |  | R | TGGTCGAGGCTGTTGGAACTGC |
| CMV 3’NTR |  | F | GTGAACGGGTTGTCCATCCAGCT |
|  |  | R | CACCCGTACCCTGAAACTAGCACG |
